# Validation of Novel Biomarkers for Prostate Cancer Progression by the Combination of Bioinformatics, Clinical and Functional Studies

**DOI:** 10.1371/journal.pone.0155901

**Published:** 2016-05-19

**Authors:** Saeid Alinezhad, Riina-Minna Väänänen, Jesse Mattsson, Yifeng Li, Terhi Tallgrén, Natalia Tong Ochoa, Anders Bjartell, Malin Åkerfelt, Pekka Taimen, Peter J. Boström, Kim Pettersson, Matthias Nees

**Affiliations:** 1 Department of Biotechnology, University of Turku, Turku, Finland; 2 Department of Clinical Sciences, Div. of Urological Cancers, Lund University, Skåne University Hospital, Malmö, Sweden; 3 Turku Centre for Biotechnology and Department of Cell Biology and Anatomy, Institute of Biomedicine, University of Turku, Turku, Finland; 4 Department of Pathology, University of Turku and Turku University Hospital, Turku, Finland; 5 Department of Urology, Turku University Hospital, Turku, Finland; University of Kentucky College of Medicine, UNITED STATES

## Abstract

The identification and validation of biomarkers for clinical applications remains an important issue for improving diagnostics and therapy in many diseases, including prostate cancer. Gene expression profiles are routinely applied to identify diagnostic and predictive biomarkers or novel targets for cancer. However, only few predictive markers identified *in silico* have also been validated for clinical, functional or mechanistic relevance in disease progression. In this study, we have used a broad, bioinformatics-based approach to identify such biomarkers across a spectrum of progression stages, including normal and tumor-adjacent, premalignant, primary and late stage lesions. Bioinformatics data mining combined with clinical validation of biomarkers by sensitive, quantitative reverse-transcription PCR (qRT-PCR), followed by functional evaluation of candidate genes in disease-relevant processes, such as cancer cell proliferation, motility and invasion. From 300 initial candidates, eight genes were selected for validation by several layers of data mining and filtering. For clinical validation, differential mRNA expression of selected genes was measured by qRT-PCR in 197 clinical prostate tissue samples including normal prostate, compared against histologically benign and cancerous tissues. Based on the qRT-PCR results, significantly different mRNA expression was confirmed in normal prostate versus malignant PCa samples (for all eight genes), but also in cancer-adjacent tissues, even in the absence of detectable cancer cells, thus pointing to the possibility of pronounced field effects in prostate lesions. For the validation of the functional properties of these genes, and to demonstrate their putative relevance for disease-relevant processes, siRNA knock-down studies were performed in both 2D and 3D organotypic cell culture models. Silencing of three genes (*DLX1*, *PLA2G7* and *RHOU)* in the prostate cancer cell lines PC3 and VCaP by siRNA resulted in marked growth arrest and cytotoxicity, particularly in 3D organotypic cell culture conditions. In addition, silencing of *PLA2G7*, *RHOU*, *ACSM1*, *LAMB1* and *CACNA1D* also resulted in reduced tumor cell invasion in PC3 organoid cultures. For *PLA2G7* and *RHOU*, the effects of siRNA silencing on proliferation and cell-motility could also be confirmed in 2D monolayer cultures. In conclusion, DLX1 and RHOU showed the strongest potential as useful clinical biomarkers for PCa diagnosis, further validated by their functional roles in PCa progression. These candidates may be useful for more reliable identification of relapses or therapy failures prior to the recurrence local or distant metastases.

## Introduction

Prostate cancer (PCa) remains a major public health problem in all western countries. PCa represents the most commonly diagnosed tumor entity after skin cancer in men, and is the second most frequent cause of cancer-related death in the United States. In the US, 233,000 new cases of PCa were diagnosed, and 29,480 men died of the disease in 2014 [1]. One in seven men may develop invasive prostate cancer during their lifetime [1]. Measurements of serum prostate-specific antigen (PSA) levels, followed by digital rectal examination (DRE) and histological examination of prostate biopsies, are still the most widely used routine diagnostic methods for PCa. In 1986, PSA was approved as a biomarker for monitoring and follow-up of PCa patients by the US Food and Drug Administration [2]. Early studies have reported a significant decrease in the mortality rate of PCa patients [3] after broad introduction of PSA tests for large-scale population screening. However, more recent studies have revealed that PSA screening can lead to widespread over-diagnosis and costly over-treatment of patients with indolent cancers [4]. As PSA is a tissue-specific, but not cancer-specific marker for the prostate, elevated levels of PSA during and after chemotherapy or radical prostatectomy indicate failure of therapy, and apparent recurrence of the disease. Although an incontestable clinical follow-up marker, PSA levels only rise after PCa remission and when progression has already occurred. Accordingly, PSA has little diagnostic and practically no predictive value for disease progression to locally advanced cancer (< 10% of the patients), or even metastatic PCa. Recently however, a panel of four kallikreins, in combination with PSA-aided risk stratification, was shown to be useful in identifying men in their fifties with a highly increased risk for disease progression and development of distant metastasis. This also provides a means to reduce the over-diagnosis of indolent disease [5]. More sensitive, informative, disease- and cancer-specific biomarkers would be necessary, in order to distinguish patients at high risk from those with indolent cancers or even premalignant precursor lesions. This may also include markers that indicate cancer-associated “field effects”, which were only recently described in prostate cancer [6] but have now become a recurrent observation that is likely to become important for diagnostic purposes. Tissue-based marker panels may have the potential to significantly improve diagnostics, resulting in more precision and earlier detection, or they may indicate the frequently observed multifocal nature of the disease [7]. Emerging applications of different ‘omics’ technologies such as genomics, transcriptomics proteomics and metabolomics have promoted the field of biomarker discovery significantly. Markers that are functionally involved in various stages of disease progression or metastatic spread might have the highest potential to successfully predict failures of radiation and anti-hormone therapies, which lead to highly aggressive and metastatic, castration-resistant prostate cancer (CRPC). Such mechanistically involved markers may not only significantly improve PCa management in clinical practice; they may also represent novel targets for therapeutic intervention. In clinical practice, the application of more predictive, tissue-based markers could be meaningfully combined with other high-risk parameters such as positive extracapsular or seminal vesicle invasion, advanced tumor stages (> T2c, T3 or T4), high Gleason grades (> 8), or high to very high PSA level (> 20 ng/ml); and would be further supported by advanced imaging technologies such as MRI.

In recent years, molecular techniques such as microarray gene expression profiling and next-generation sequencing (NGS) have greatly facilitated genome-wide studies of tumor gene expression profiles. Accordingly, microarray and NGS-based gene expression profiling has been widely used for identifying panels of prognostic [8] and predictive biomarkers, including such that may be indicative for PCa recurrence [9,10], early and late stages of cancer progression, or field effects. Identification of novel, informative biomarkers for lung and colorectal cancer by systematic mining of public gene expression datasets such as The Cancer Genome Atlas (TCGA) has been reported [11,12] elsewhere. The TCGA database contains many large-scale gene expression studies based on clinical PCa biopsies [13,14,15], derived from publicly funded research and openly available datasets. The cBio Cancer Genomics Portal [16] (http://www.cbioportal.org) is the main open-access resource for mining the TCGA cancer genomics data sets, and currently hosts more than 21.000 tumor samples from > 20 different cancer entities and 91 studies. We have selected one of the largest and most comprehensive of these microarray expression studies on PCa’s, performed mainly on clinical biopsies, conducted by Taylor et al. at Memorial Sloan-Kettering Cancer Center (MSKCC) [13] in 2010. This study includes 181 primary PCa samples, 37 metastatic PCa samples, 12 PCa cell lines and xenografts, and 29 normal prostate tissues as healthy controls. A large number of pathological and clinical parameters such as Gleason grades, TNM stages, positive surgical margins, the status of local invasion of cancer cells into lymph nodes, seminal vesicles, or extracapsular space, or distant metastasis are annotated. These parameters are deemed most important to assess PCa aggressiveness, and reliably predict poor outcome of the disease. Our specific goal for data mining was to utilize an open, unbiased approach, addressing a broad spectrum of normal tissues, primary cancers, and late-stage, advanced lesions. Since the goal was not to predict the clinical outcome or assess the individual risk for patients or patient subgroups based on mRNA expression data, the data were not split into training and test sets for cross-validation. We also did not aim for the evaluation of a “predictive marker signature” to be used across other, independent mRNA gene expression studies. Cross validation was therefore not essential in order to confirm how statistically accurate a set of predictive biomarkers would perform in additional data sets. In contrast, our aim was to identify previously unreported biomarkers and validate them across the entire spectrum of clinical biopsies available from PCa. Our approach was based on analysis of differential mRNA expression for the identification of biomarker candidates, followed by their experimental correlation with the most critical clinical parameters in independent sample collections, which were often of very different origin compared to the microarray data. The main focus was on the functional validation of biomarkers in relation to PCa initiation and progression, ideally combined with evaluating their potential diagnostic value.

For independent validation of the initial findings, we mined additional resources, such as the *in silico* transcriptomics (IST) database [17] (http://ist.medisapiens.com). This database includes mRNA gene expression data from over 20.000 Affymetrix microarrays, covering 60 healthy tissues, 104 malignant and 64 other disease types. For data mining, we have utilized Ingenuity Pathway Analysis (IPA), which provides gene association and ontology information, and allows filtering of genes based on functional aspects. Last not least, we used the Pubmed literature information system to filter out biomarkers that have been repeatedly described before as associated with PCa. A batch mode text mining tool (http://pmid.us) was used, which allowed sca1nning through the entire literature for the mesh heading “prostate cancer”, against co-occurrence of hundreds of candidate genes entered as “gene symbols”. With this strategy, a set of 300 putative biomarker candidates was prioritized step by step, using a combination of different data and text mining or filtering approaches, highlighting markers that were most strongly correlated with general aspects of PCa progression, therapy failure, or progression to metastatic CRPC, but not previously covered by a large body of scientific reports. Eight genes were selected for clinical and functional validation. For this purpose, quantitative, internally standardized real-time reverse-transcription PCR (RT-PCR) was applied, utilizing four independent tissue sample collections from radical prostatectomy and cystoprostatectomy. These contained normal cystoprostatectomy samples, histologically benign tissue from cystoprostatectomy specimens with incidental prostate cancer, in addition to histologically benign tissues, and malignant cancer from radical prostatectomy specimens.

Recent advances in cell biology have facilitated systematic functional validation studies (functional genetics) of biomarker candidates, based on effective approaches such as small interference RNA (siRNA or RNAi), CRISPR/Cas9 and TALEN technologies. Of these, siRNA studies remain the most accessible, affordable and fastest technologies in experimental practice, and represent the primary approach in functional target validation. In order to explore functional effects of selected genes on growth, proliferation and invasive properties of prostate cancer cells, siRNA knock-down studies for selected genes were performed in both 2D and organotypic 3D models (organotypic cell cultures) using the poorly invasive VCaP and the highly aggressive PC3 cell lines.

## Materials and Methods

### Analysis of gene expression profiling to identify candidate biomarkers

**Fig 1:**
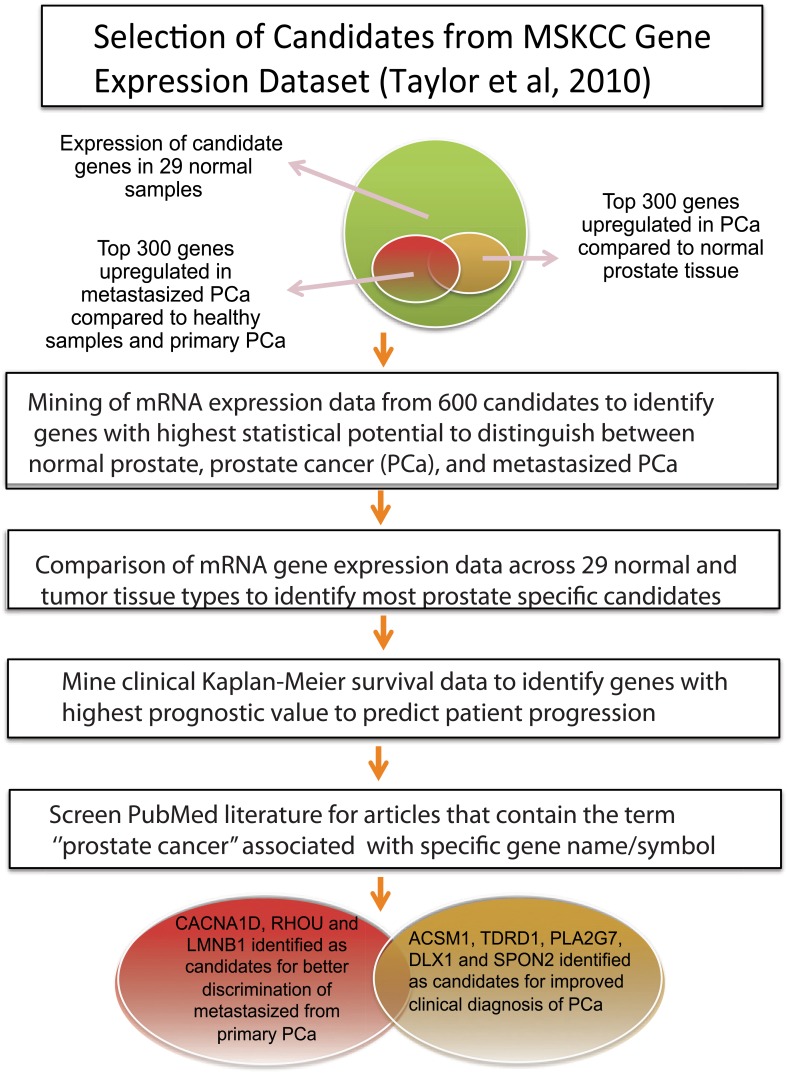
Schematic illustration of different bioinformatics analyses and filtering methods that applied to mine large-scale gene expression profiling datasets and to select the most interesting biomarkers for subsequent clinical and functional validation.

A panel of different bioinformatics analysis and filtering methods was applied to mine large-scale gene expression profiling datasets, and to select the most informative, putative biomarkers for prognosis and/or monitoring of disease progression (summarized in Fig 1). We used Bioconductor/R-based data normalization and the RMA (Robust Multi-chip Average) package for processing and normalization of Affymetrix experiment sets extracted from the TCGA/cBio portal. Next, genes were ranked according to differential gene expression across the main sample groups (N, normal; T, primary PCa; and M, metastatic PCa). For this purpose, we used several statistical tests for significance (ANOVA, T-test or Z-score and the Mann-Whitney U Test; and then filtered candidates by multiple testing corrections (Bonferroni). In parallel, we used the SAM or “Significance Analyses for Microarrays” program, using False Discovery Rate (FDR) criteria for gene selection. This approach resulted in overlapping, similar gene sets (data not shown). We restricted our analysis to the large MSKCC dataset (218 tumor profiles, including 37 metastases, 12 PCa cell lines, and 29 normal prostate tissues). Differential expression of each gene across 181 primary cancer samples was compared to the 29 normal samples (*T versus N comparison*). Similarly, expression in the 37 metastatic samples was compared to the 181 primary PCa samples (*M versus T*). The average fold change of mRNA expression and statistical significance across all samples were calculated, and genes were ranked according to the strongest differences in mRNA gene expression (fold change, see S1 and S2 Files). The top 300 hits in each group (T vs. N, and M vs. T) were then subject to additional data filtering: Only those genes were selected for follow up that showed the statistically most significant, robust and reproducible differences between normal, primary PCa and metastatic PCa samples (based on FDR estimations or Bonferroni-filter). We also used the Ingenuity Pathway Analysis (IPA) software tool for additional filtering options of the original gene lists, based on both statistical parameters as well as functional “gene ontology” and mechanistic pathway annotations. We utilized the ranked list of candidates generated by IPA for additional correlation with clinical parameters (Kaplan-Meier plots), assessment of tissue-specific expression, and literature text mining (next paragraphs). For cancer-specific genes identified as differentially expressed between the N and T-groups, ranking was higher if differential mRNA gene expression was also observed between the T and M subcategories.

**Fig 2:**
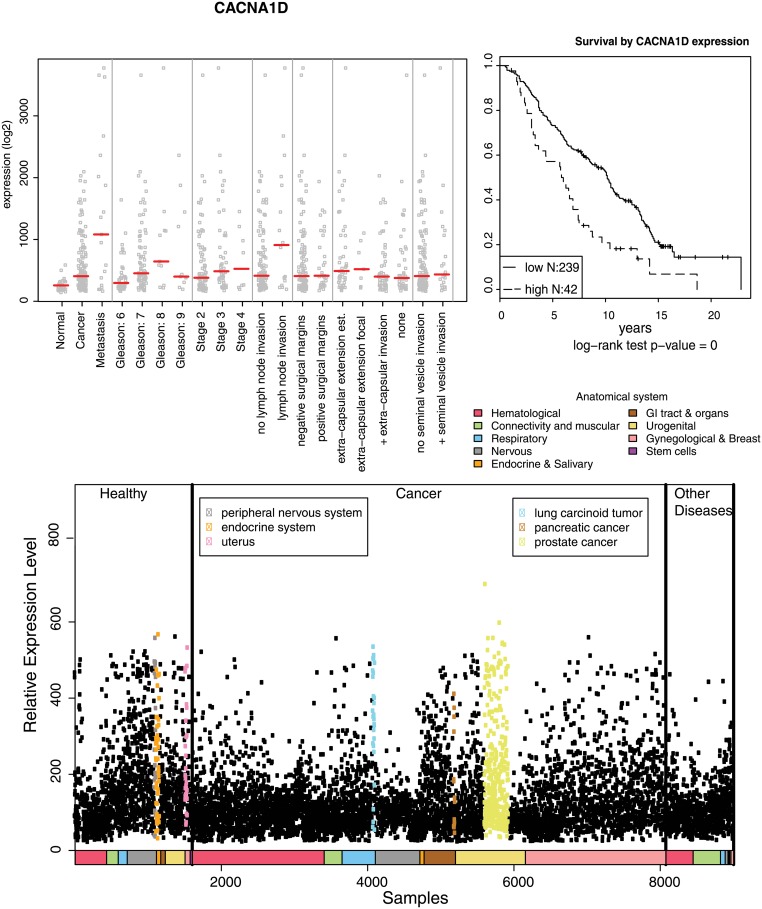
Representation of the most important data format, plots and visualization results, used in data mining and prioritization of candidate biomarkers, using the CACNA1D gene as an example. A) Dot plot chart illustrating the association of mRNA gene expression of CACNA1D in normal and cancer samples with a panel of the most informative clinico-pathological parameters. B) Kaplan- Meier analyses, showing the correlation of high levels of mRNA expression for CACNA1D with reduced overall survival analysis for PCa patients. C) Relative expression of CACNA1D across a panel of 60+ normal and cancer-related human tissue types and diseases.

For each selected gene, box-plot expression graphs were generated using an in-house html-based data visualization tool (R-executable (REX)). This allows systematic, user-driven data mining of datasets according to clinical parameters (e.g. tumor subgroups such as primary vs. metastatic cancers, lymph node and extracapsular invasion, infiltration of surgical margins, and distant metastasis). In addition, differential expression between low (4–6), intermediate (7) and high (8–10) Gleason scores, or between different stages of the disease (> T2, T3 or T4), and disease categories such as primary vs. lymph node metastasis, were systematically assessed (Fig 2A). Next, we prioritized genes that were specifically, preferentially or strongly expressed in prostate compared to all other human tissues, including other tumor entities. For this purpose, tissue-specific expression scores provided by the IST online database were used (Fig 2C). The tissue-specific expression scores and box-plot expression graphs for other selected genes are summarized in S3 File.

Next, we addressed clinical outcome, but used other data sets in addition to the MSKCC microarray data. For each selected gene, a Kaplan-Meier patient outcome plot based on an independent, large-scale clinical study with publicly available data [15] was generated. For this purpose, we focused on total survival and disease-free intervals, using non-parametric statistics that discriminate patient survival functions from clinical lifetime data (Fig 2B). Since approximately 85–90% of patients even with locally advanced PCa show long-term survival (> 5 or 10 years) and mostly good clinical outcome, and only 10–15% of these patients progress to CRPC, we have chosen equivalent numbers also for the Kaplan-Meier analyses. Accordingly, 85% of the patients were grouped in the “low risk/good clinical outcome” category, compared to 15% of high-risk patients that progress to advanced PCa.

Finally, to prioritize novel candidate biomarkers for subsequent functional and clinical validation, our list of biomarker candidates was filtered against the entire PubMed literature database. Co-occurrence of candidate gene names and HUGO symbols with the term “prostate cancer” in the PubMed database entries was screened systematically, using the Pubmed batch-search tool (http://pmid.us). Genes that were mentioned more than five times in indexed scientific publications were automatically excluded as “previously described in detail” (Status of literature search: September 2012). These last filtering steps led to eight genes for experimental validation. Gene expression plots from IST database and dot plots from the MSKCC data set are summarized in S3 File. We aimed to identify and confirm general PCa-specific biomarkers (diagnostic markers), including biomarkers that may indicate early and multifocal disease stages or “field effects”. In addition, we wanted to validate potential PCa progression markers (prognostic markers) that might indicate the development of relapse, CRPC and metastatic lesions after therapy failure.

### Tissue samples for clinical biomarker validation

The Ethics Committee of the Hospital District of Southwest Finland approved the study protocol. It was in accordance with the Helsinki Declaration of 1975, as revised in 1996, with written informed consent obtained from each participant. Two independent sets of prostate-related tissue samples were used for clinical validation. The first set consisted of 180 prostate tissue samples obtained from a total of 90 primary PCa patients, operated by radical prostatectomy (RP) in Turku University Hospital (TYKS). From 90 patients, only 2 patients had received HRH-analogues before the surgery as preoperative treatment. From each prostate, two tissue samples were taken. One was derived from the suspected cancerous area, the other from the adjacent area, suspected to be benign. The histo-pathology of half of each tissue sample was examined by TYKS pathologists, while the other half was immediately stored in guanidine isothiocyanate (GITC) buffer for RNA extraction. Examination revealed that for 30 of the patients, both samples were taken from the benign area; for 15 patients, both samples were taken from the cancerous area; and for 45 patients, one sample was taken from benign and the other from the cancerous area (as originally intended). Only two samples needed to be excluded because of technical problems with RNA extraction. Finally, 178 samples (104 benign samples and 74 cancerous samples) were left for gene expression analysis by qRT-PCR. The clinical and pathological features of all 90 PCa cases are presented in Table 1.

**Table 1:** Characteristics of the patient cohort of 90 men with prostate carcinoma who underwent radical prostatectomy, PSA values were measured from serum preoperatively.

	Median (min, max)
Number of patients:	90
Age at surgery (years):	62 (48, 72)
Preoperative serum PSA:	7.4 (2.7, 58)
	Number of samples (percentage):
Pathological T stage/category	
pT2	51 (57%)
pT3 and pT4	35 (39%)
Unknown*	4 (4%)
Pathological Gleason scores	
≤6	43 (48%)
7	36 (40%)
≥8	7 (8%)
Unknown*	4 (4%)

* Values missing for four patients.

The second cohort included 19 prostate tissue samples, obtained from patients with bladder cancer without any clinical symptoms of PCa. These were operated by cystoprostatectomy (CP) at Skåne University Hospital in Malmö, Sweden. Experienced pathologists, using the same protocol as previously described for the RP specimens, examined all prostate specimens. 12 of the 19 prostate specimens in this collection had incidental PCa (IPCa), while seven were free of any detectable carcinoma foci. In the case of IPCa, tissue samples for gene expression analysis were taken from the benign area.

### Extraction and reverse transcription of RNA

Extraction of RNA from tissue samples has been previously described [18]. Briefly, the RNeasy Mini Kit (Qiagen, Germany) was used according to manufacturer’s instructions. During RNA extraction, and after the initial cell lysis step, a known amount of RNA of an artificially mutated *KLK3* gene, termed mmPSA, was added to the sample as an internal control and for absolute quantification of expression levels. The quality of the mRNA obtained was verified by agarose gel electrophoresis, and the final RNA concentration was measured by a NanoDrop N2000 spectrophotometer (NanoDrop Technology, LTD Lab). Reverse transcribed cDNA was generated using the High Capacity cDNA Archive Kit (Applied Biosystems, USA), following the manufacturer’s instructions.

### Quantitative real-time RT-PCR

Our previously described protocol [19] for hydrolysis-enhanced luminescent chelate chemistry, based on time-resolved fluorometry, was used also here for quantitative real-time RT-PCR. For each gene, a pair of specific primers, and matching reporter and quencher probes were designed and purchased from Thermo Fisher (Germany) (S1 Table). To label the reporter probes effectively with a nonadentate europium chelate, a previously described procedure [20] was used. Real-time PCR was performed in a total volume of 25 μL, containing 2.5 μL of template cDNA with Hotmaster^™^ Taq DNA polymerase (5 Prime, Germany) or AmpliTaq^®^ Gold DNA polymerase (Applied Biosystems, USA). Samples were run in triplicates. To correct for potential RNA degradation and the amount of RNA lost during extraction and reverse transcription, the obtained transcript level data need to be normalized. Housekeeping genes (e.g. *GAPDH*) are assumed to be transcribed at a constant level in different tissues and in all conditions, and are widely used for normalization of RT-PCR results in relative quantification methods [21]. However, the reliability of housekeeping genes for normalization has often been questioned due to their instability during storage and inconsistent expression changes in different disease conditions [22,23]. Addition of a fixed amount of an artificial RNA, which is not expressed in the specimens inherently as internal reference RNA to samples (before extraction) is considered as more reliable alternative for normalization [24,25]. In this study, we have taken the advantages of using artificial RNA (mmPSA) for normalization and the benefits of using probes labeled with lanthanide chelates instead of Taqman probes, which enable the time-resolved fluorometry for qRT-PCR assays.

### Statistical analyses

For qRT-PCR analyses, the samples were considered positive if all three replicates were above the lowest assay detection limit (LDL). The absolute, numeric quantity of gene expression for any gene of interest was calculated by using internal standard values, normalized by the amount of total RNA used in these assays. To test the differences of transcript levels of candidate genes between case and control samples as well as between different sample categories, the non-parametric Mann-Whitney *U* test was applied. To evaluate the performance of each candidate gene in a diagnostic setting, a receiver-operating characteristic (ROC) curve analysis was performed and the area under the curve (AUC) used to assess sensitivity and specificity of the assays. To identify the association of mRNA transcript levels of a particular candidate gene with tumor progression (measured as PSA relapse in the clinics), the RP samples were divided in two groups, reflecting PSA relapse and No relapse, respectively; according to clinical follow-up data. Any association between candidate gene expression levels and clinical or pathological parameters such as Gleason grade, Gleason score, the percentage of cancer versus stromal cells in tissue biopsies, and clinical T category (T-stage) was addressed by the non-parametric Mann-Whitney *U* test, in order to identify statistically significant differences (p<0.05). Statistical analyses were performed with the SPSS software, version 22 (IBM, USA).

### Functional gene knock-down studies with siRNAs

PC-3 androgen-independent human prostate cancer cells from bone-metastasized prostate adenocarcinoma were purchased from ATCC (USA) [18] and grown in RPMI-1640 medium at 37°C in standard cell culture conditions (95% humidity and 5% CO2). For siRNA knock-down studies, a total of 31 different siRNAs were ordered from Qiagen (Germany) (three different siRNAs for *ACSM1* and four different siRNAs for each of the other seven genes). We used several PCa cell lines, including PC-3, VCaP and LNCaP cells, for these functional validation experiments. To achieve the most efficient possible knock-down for each gene, several siRNAs were tested both individually as well as in pooled mixtures containing all three or four siRNAs at equal concentrations. ALLStars Hs cell death control siRNA (Qiagen, Germany) was used as a positive control to estimate the efficacy of transfection in each experiment. The siRNAs were pre-loaded into wells, Hiperfect transfection agent (Qiagen, Germany) in Opti-MEM medium (Invitrogen, USA) was added, and incubated for 15 minutes at room temperature. Finally, 2000 to 5000 PC-3, LNCaP or VCaP cells were added into each well. The final concentrations of siRNA and Hiperfect in each reaction were 4 nM and 8 nM, respectively. To evaluate the efficiency of RNA interference for each gene with the different siRNAs, total RNA was extracted from transfected cells five days after siRNA transfection and specific mRNA expression of the target genes was measured by qRT-PCR. To select those siRNA molecules that result in the most effective knock-down, normalized expression values for each treated sample were divided by the expression level of the candidate gene in untreated control cells (“scrambled control”). To exclude any possible effects of this procedure on target gene expression, mock-transfected cells (using only transfection agent) were included as a second control.

### Fluorescent assays to measure apoptosis and growth inhibition of VCaP organoids after siRNA transfection

VCaP cells failed to grow in 3D conditions, if directly embedded into Matrigel or collagen cultures. Therefore, VCaP cells were transfected with siRNAs as described for PC3. Next, transfected VCaP cells were grown in round bottomed plates. The cells were allowed to aggregate and form spheroids for seven days after transfection. A small number of spheroids (1–6) were transferred into 3D culture plate wells. Such VCaP organoids showed few phenotypic effects, but displayed altered organoid growth and different levels of apoptotic, dead or dying cells in 3D culture in response to siRNAs, thus were considered informative. Cell viability was determined with CellTiter-Glo assay according to the manufacturer’s instructions (Promega, USA). Briefly, CellTiter-Glo buffer and lyophilized substrate was combined, 100 μl of said solution is added to each sample well and the plate is shaken for 30 minutes. The luminescence which results from cell lysis and which are in proportion with the amount of adenosine triphosphate (ATP), was measured with an EnVision multilabel plate reader (Wallac, Finland). Furthermore, the NucView caspase 3/7 assay has been used which detects cells actively undergoing programmed cell death (described in detail in [26,27,28].

### 2D cell migration and invasion assay

The invasive or migratory potential of the PCa cells was investigated with a “scratch wound” or wound-healing assay, performed in 96-well ImageLock plates (Essen Bioscience, USA). Both VCaP and LNCaP cells lacked invasive or motile properties in 2D or 3D conditions. These cell lines did not show wound closure properties, and were not useful in scratch wound assays. For this reason, we used PC3 cells, which also expressed seven of the eight candidate genes at significant levels, were straightforward to transfect by siRNAs, and also showed marked motile/invasive properties in both 2D and 3D cell culture conditions. PC-3 cells were transfected with the different individual siRNAs (as described above), and plated onto the wells after transfection. When the cells reached confluence, which occurred roughly 72 hours after transfection (2–3 population doublings), a fixed-width wound was applied, using the Woundmaker device (Essen Bioscience, USA). This approach provides uniform, standardized experimental settings across all wells of an entire 96-well multititer plate, and facilitates a reliable quantitative evaluation of cell motility in each replicate. After wounding, wells containing a “scratched” monolayer were carefully washed once with PBS to remove floating, dislocated cells and cell debris. Fresh cell culture medium was then added to the wells, and the closure of the wound boundaries was quantified and monitored for up to 72 h with the IncuCyte live-cell imager (Essen Bioscience, USA). Images were acquired at 1h intervals, and analyzed using the automated Cell Migration software module provided with the Essen IncuCyte imager. Kinetic, time-course plots of wound closure were visualized in “micro-plate view” format, and multiple export metrics such as relative wound density, wound confluence and wound width were calculated as a measure for cell migration, and to identify any quantitative changes to cell motility after siRNA knock-down.

### Organotypic 3D cell culture models and image acquisition for morphological analyses

As described for the 2D wound healing experiments, we investigated the functional effects of siRNA knock-down of candidate genes in a panel of PCa cell lines, and used LNCaP, VCaP and PC3 cell lines for this purpose. LNCaP cells were difficult to transfect, did not express all eight of the target genes, and failed to form overtly invasive structures in 3D culture. Furthermore, VCaP organoids did not readily grow when embedded into Matrigel, but had to be grown in 3D round bottom culture plates, where few morphometric differences became apparent. To monitor for changes in dynamic properties such as invasiveness or tumor-specific differentiation patterns, we therefore focused mainly on PC3 cells. PC-3 cells are ideal for the systematic analysis of morphologic, differentiation-related features and to evaluate the impact of siRNA silencing on epithelial tumor cell plasticity. Embedded in Matrigel, PC-3 organoids show a characteristic, disturbed differentiation capacity: initially, mature, acinar organoids form that resemble low-grade, well-differentiated tumors and lack any invasive features. After a spontaneous morphological conversion (during days 8–11 in 3D culture), PC3 organoids form heavily invasive characteristic for advanced, metastatic CRPC tumors. Experimental protocols for siRNA knock-down in 3D cell culture conditions were described elsewhere in detail [29]. Briefly, PC-3 cells were transfected only with the most efficient, single siRNAs, as described above. Transfection efficacy was evaluated three days after transfection by quantitative qRT-PCR. 72 hours after transfection, cells were detached and transferred to uncoated Angiogenesis slides (Ibidi GmbH, Germany), as described elsewhere [26,27,28]. Single, siRNA-transfected cells were embedded between two layers of Matrigel (500–1000 cells/well), resulting in an average cell density of approximately 1800 cells/cm^2^. Under these conditions, single PC3 cells typically give rise to a single, proliferating tumor organoid. Maintenance of siRNA knock-down even > 10 days after transfer into 3D cultures was validated in a previous study [29]. After ten days in 3D culture, the resulting tumor organoids cells were stained using a live/dead labeling strategy: Calcein AM live cell dye (ThermoFisher, USA) is actively incorporated and accumulates in living cells, while and ethidium homodimer 1 (EthD-1) stains only dead and apoptotic cells that lack intact plasma membranes. Stacks of confocal images were taken with an Axiovert-200M microscope (Zeiss, Germany), using a Yokogawa spinning disc confocal unit and a 5X Plan-Neofluar objective. A Z-stack of twenty focal images, with a step-size (distance) of 20–35 μm between layers, was acquired. SlideBook (Intelligent Imaging Innovations Inc., USA) was used to capture composite images and to create intensity projections. Next, the ImageJ software (NIH, USA) was used for initial image normalization and to remove background and noise. The Automated Morphometric Image Data Analysis software package (AMIDA, University of Turku) was used for analysis of images and quantitative measurements of morphological changes in three-dimensional tumor organoids formed by PC-3 cells. The AMIDA software is publicly available, and basic operations and concepts have been previously described [28].

### Morphometric and phenotypic parameters for 3D image analyses

Strong cell-cell interactions between tumor cells lead to mature organoids, often surrounded by a basement membrane (BM) or basal lamina. Such organoids are also formed by PC3 cells, and recapitulate key parts of the epithelial differentiation program of normal and low-grade PCa cells. Mature organoids are typically round, smooth and lack invasive processes. In contrast, advanced and aggressive PCa cells form irregular, rough and rapidly growing organoids, devoid of a functional BM; and may show pronounced invasive features. According to these different morphologies, a total of 26 different parameters were evaluated, focusing mainly on 3D organoid growth, differentiation/maturation, and invasive properties. The **Area** parameter of segmented organoids (measured as number of pixels) is a simple measure of cell growth and proliferation, and correlates with the number of cells within organoids. Cytotoxic effects typically result in reduced **Area** measures; they often also lead to increased **Density** and less invasive structures (increased **Roundness**).

The **AppIndex**, **MaxApp** and **Roughness** functions are measures for invasive processes observed in 3D cultures. Silencing of genes can affects invasive properties, and alter the number and degree of **MaxApp** values (# of invasive protrusions/organoid), often without affecting proliferation (**Area** function). Invasive properties are negatively correlated to the **Roundness** measure, which represents a measure for epithelial differentiation, maturation, formation of a functional BM and strong cell-cell contacts. Furthermore, the **Density** function positively correlates with well-differentiated 3D structures, but negatively with invasiveness. In practice, effects resulting in growth inhibition often coincide or overlap with anti-invasive effects, and both can co-occur frequently.

## Results

### Analysis of differential mRNA gene expression profiles and identification of putative marker genes

Eight genes (*ACSM1*, *TDRD1*, *PLA2G7*, *SPON2*, *DLX1*, *CACNA1D*, *RHOU*, and *LMNB1*) were selected by bioinformatics data mining, based on their overexpression in primary PCa compared to normal tissue samples mainly in the MSKCC data set; and expression was confirmed in other data sets such as the IST database. Three of the candidates (*CACNA1D*, *RHOU*, and *LMNB1*) showed potency to discriminate metastatic PCa from primary PCa. One gene (*PLA2G7*) has been previously identified by our laboratory as a putative diagnostic marker for PCa progression, in connection with the TMPRSS2-ERG fusion gene status. *PLA2G7* was suggested as a functionally important drug target, in particular in ERG-positive PCa [30,31]. In this study, *PLA2G7* was included as a reference gene for both internal validation purposes and optimization of functional assay development. The genomic location and physiologic function of the candidate biomarkers are listed in Table 2.

**Table 2:** Genomic location and physiologic function of the candidate biomarkers.

Genes name	Genomic location*	Function^+^
*ACSM1* (Acyl-CoA Synthetase Medium-Chain Family Member 1)	16p12.3	GTP binding and fatty acid ligase activity. Related pathways: Metabolism and fatty acid beta-oxidation
*CACNA1D* (Calcium Channel, Voltage-Dependent, L Type, Alpha 1D Subunit)	3p21.1	Involved in muscle contraction, hormone or neurotransmitter release, and gene expression. Related pathways: MAPK signaling pathway and Developmental Biology
*DLX1* (Distal-Less Homeobox 1)	2q31.1	Function as a transcriptional regulator of signals from multiple TGF-{beta} superfamily members. Related pathways: Regulation of nuclear SMAD2/3 signaling and Packaging Of Telomere Ends
*LMNB1* (Lamin B1)	5q23.2	Involved in nuclear stability, chromatin structure and gene expression. Related pathways: Cell Cycle, Mitotic and Cytoskeletal Signaling
*PLA2G7* (Phospholipase A2, Group VII)	6p12.3	Hydrolyze phospholipids into fatty acids and other lipophilic molecules. Related pathways: Apoptotic Pathways in Synovial Fibroblasts and Activation of cAMP-Dependent PKA
*RHOU* (Ras Homolog Family Member U)	1q42.13	Regulation of cell morphology, cytoskeletal organization, and cell proliferation. Related pathways: Signaling by GPCR and Signaling by Rho GTPases
*SPON2* (Spondin 2, Extracellular Matrix Protein)	4p16.3	Cell adhesion protein that promotes adhesion and outgrowth of hippocampal embryonic neurons. Related pathways are Phospholipase-C Pathway and ERK Signaling
*TDRD1* (Tudor Domain Containing 1)	10q25.3	Plays a central role during spermatogenesis by participating in the repression transposable elements and preventing their mobilization. Related pathways: Gene Expression and Mitotic Prophase

* Based on genomic locations by Ensembl (http://www.ensembl.org)

^+^ Based on GeneCards Human Gene Database (http://www.genecards.org).

### Clinical validation of differential gene expression for selected candidate genes by quantitative real-time RT-PCR

To investigate the upregulation of the selected eight candidate biomarker genes in cancer, the mRNA expression of the eight genes was measured by quantitative real-time RT-PCR. To identify genes that could discriminate between different histological tumor subtypes, all tissue samples analyzed were assigned into four sample groups: 1. cystoprostatectomy (CP) samples with no cancer foci (CP-B), 2. cystoprostatectomy samples with incidental PCa (CP-IPCa), 3. histologically benign radical prostatectomy samples (RP-B) and 4. malignant radical prostatectomy samples (RP-PCa). Expression of *ACSM1*, *CACNA1D*, *PLA2G7* and *SPON2* was detected in all tissue samples across different groups of samples. However, the expression of *DLX1* was detected in 73/74 (99%) of the RP-PCa samples, but could not be detected in any of the seven CP-B samples. Detection of genes in various sample groups is outlined in Table 3. The levels of differential expression between each set of two sample groups and across different clinic-pathological parameters were analyzed by the non-parametric Mann-Whitney U test (Table 4).

**Table 3:** Frequency of detection of target mRNAs in benign prostate tissue from patients without PCa (CP-B samples) and with incidental PCa (CP-IPCa samples) and in histologically benign (RP-B samples) and cancerous (RP-PCa) tissue of patients with PCa.

Number and percentage of samples in which target mRNA could be detected				
Target mRNA	CP-B samples (n = 7)	CP-IPCa samples (n = 12)	RP-B samples (n = 104)	RP-PCa samples (n = 74)
***LMNB1***	5 (71%)	12 (100%)	104 (100%)	74 (100%)
***ACSM1***	7 (100%)	12 (100%)	104 (100%)	74 (100%)
***CACNA1D***	7 (100%)	12 (100%)	104 (100%)	74 (100%)
***RHOU***	2 (28%)	7 (58%)	104 (100%)	74 (100%)
***DLX1***	0 (0%)	5 (41%)	88 (85%)	73 (99%)
***TDRD1***	2 (28%)	6 (50%)	101 (97%)	72 (97%)
***PLA2G7***	7 (100%)	12 (100%)	104 (100%)	74 (100%)
***SPON2***	7 (100%)	12 (100%)	104 (100%)	74 (100%)

**Table 4:** Association of mRNA expression of selected target genes in prostate tissues, and statistical correlation with major clinical and pathological parameters of PCa biopsies, calculated with the Mann-Whitney test.

	Target mRNA								
	*KLK3*	*RHOU*	*ACSM1*	*CACNA1D*	*LMNB1*	*TDRD1*	*PLA2G7*	*DLX1*	*SPON2*
**CP (n = 19) vs. RP (n = 178)**	0.001	<0.001	<0.001	<0.001	<0.001	<0.001	<0.001	<0.001	<0.001
**CP (n = 19) vs. RP-PCa (n = 74)**	0.001	<0.001	<0.001	<0.001	<0.001	<0.001	<0.001	<0.001	<0.001
**CP (n = 19) vs. RP-B (n = 104)**	0.008	<0.001	<0.001	<0.001	0.001	0.001	<0.001	0.008	<0.001
RP-B (n = 104) vs. CP-IPCa (n = 12)	0,22	0.05	<0.001	0.007	0.02	0.11	<0.001	0.53	0.003
CP-IPCa (n = 12) vs. CP-B (n = 7)	0.34	0.15	0.31	0.31	0.12	0.15	0.8	0.056	0.310
**RP-PCa (n = 74) vs. RP-B (n = 104)**	0.9	0.30	0.001	0.001	0.14	<0.001	0.804	<0.001	0.00
PCa Gleason ≥ 7 (n = 43) vs. Gleason ≤,6 (n = 43)*	0.866	0.249	0.849	0.799	0.331	0.115	0.048	0.074	0.707
pT 2 (n = 51) vs. pT 3,4 (n = 35)*	0.161	0.535	0.833	0.004	0.072	0.377	0.715	0.001	0.775
**PSA relapse (n = 15) vs. No PSA relapse (n = 65)** *	0.225	0.980	0.206	0.090	0.151	0.209	0.427	0.156	0.023

* For patients represented by two samples, a single value of mRNA expression of each gene for each patient was chosen, consistently from the right lobe of the prostate. Statistically significant P values are shown in red color. CP represents cystoprostatectomy samples, RP-B represents the histologically benign radical prostatectomy samples and RP-PCa represents the cancerous radical prostatectomy samples. Gleason indicates the Gleason score of entire prostate specimen removed from the patient.

The comparison of the cancer-related sample group 4 (RP-PCa) vs. the validated benign sample group 1 (CP-B), and between all cystoprostatectomy samples (CP) versus all radical prostatectomy (RP) samples revealed statistically significant expression differences for all eight genes. Corresponding to the initial gene identification and ranking strategy, it is not surprising that the validated normal samples (CP-B) showed marked differences to all three, directly or indirectly disease-associated sample groups, even if these did not contain overt malignant tissue. In particular, the comparison of the confirmed benign cystoprostatectomy sample group 1 (CP-B) to the radical prostatectomy sample group 3 (RP-B) resulted in highly statistically significant upregulation for all eight genes, despite the fact that these biopsies did not contain histologically detectable prostate cancer. This finding points to the generalized observation that many malignancy-related marker genes may be upregulated and be detectable already in non-malignant areas, adjacent to malignant tissue. While such “field effects” have been described in head & neck or lung cancers already since the 1950, the corresponding concept in PrCa has only been recently emerging. The same basic observations apply for the comparisons of the CP-B group against the malignant samples (RP-PCa) or all radical prostatectomy samples combined (RP). As would be expected, this latter group showed higher expression patterns for most genes than cystoprostatectomy samples with small, incidental cancer.

**Fig 3:**
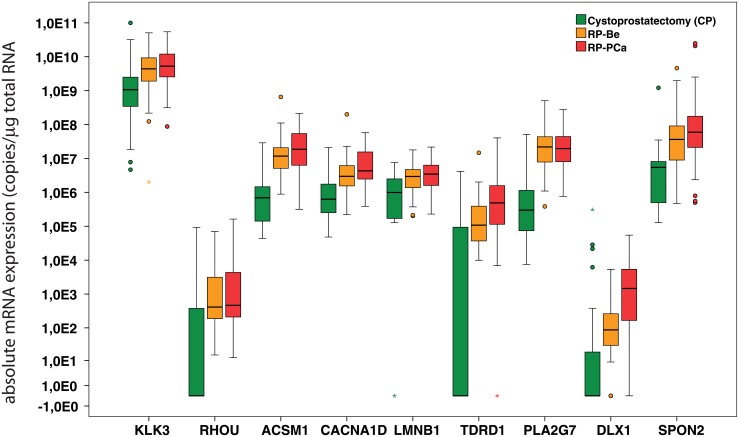
Differential mRNA expression levels for eight candidate biomarker genes and KLK3 (mRNA copy number/μg total RNA) in cystoprostatectomy samples (CP), histologically benign radical prostatectomy samples (RP-Be) and cancerous radical prostatectomy samples (RP-PCa). Boxes show the interquartile range with the line in the middle of them denoting the median value and circles represent the outliers.

When the two large groups of RP samples (benign RP-B and malignant RP-PCa) were compared with each other, most of the differences were statistically still highly significant (p≤0.001), except for *RHOU*, *LMNB1* and *PLA2G7*, indicating the stronger impact of detectable cancer tissue on biomarker expression patterns compared to likely field effects. Similarly, the comparison of histologically benign radical prostatectomy samples to incidental samples from cancerous patients (RP-B vs. CP-IPCa) revealed statistically significant differential expression (p ≤ 0.05) for six of the eight genes, with the exception of *TDRD1* and *DLX1*. Also these observations may be due to the aforementioned field effects in cancer-associated or adjacent tissues. In contrast, and probably due to the small sample size, no statistically significant differences for any of the eight genes were observed between the CP-B and CP-IPCa sample groups. Experimental results and fold-changes for all eight biomarker genes across sample groups on a logarithmic scale, in relation to KLK3 (PSA), are shown in Fig 3.

### Association of mRNA expression of the candidate biomarker genes with clinical and pathological parameters

The clinical cancer samples were divided into additional sample groups, based on Gleason scores (ranging from Gleason 4–9). No statistically significant differences for mRNA expression were observed between different single Gleason scores, probably because none of the sample groups were large enough. Thus, clinical samples were divided in two larger groups: one consisting of more aggressive (Gleason grades ≥7–9) tumors, the second of less aggressive (Gleason < 7; including Gleason 4, 5, and 6) tumors. For the patients represented by two samples, a single value of mRNA expression of each gene for each patient was chosen, consistently from the right lobe of the prostate. Applying the single value of mRNA expression of each gene for each patient resulted in significant differences for only *PLA2G7* (p = 0.048).

When using a single value of mRNA expression of each gene, and for each patient represented by two samples (consistently from the right lobe), differential expression of *CACNA1D* and *DLX1* correlated strongly with the T2 and T3 categories of TNM staging (p = 0.004 and 0.001, respectively). Next, we examined if differential mRNA expression of any of the eight genes might predict cancer recurrence or progression, and would therefore harbor specific prognostic potential. Applying the single mRNA expression value for each patient resulted in a significant difference only for the expression of *SPON2* (p = 0.023) between patients with or without PSA relapse.

Next, cancer samples from radical prostatectomies (RP-PCa) were also divided into two groups according to the content of stromal tissue within the biopsies. One group comprised all samples containing over 33% of tumor content (and < 66% stroma), the other those below 33% of carcinoma content (> 66% stroma). The expression of *DLX1* was significantly different between two groups and correlated with high tumor content, with p- values of 0.018.

### Specificity and sensitivity of biomarkers—receiver operating curves (ROC)

**Fig 4:**
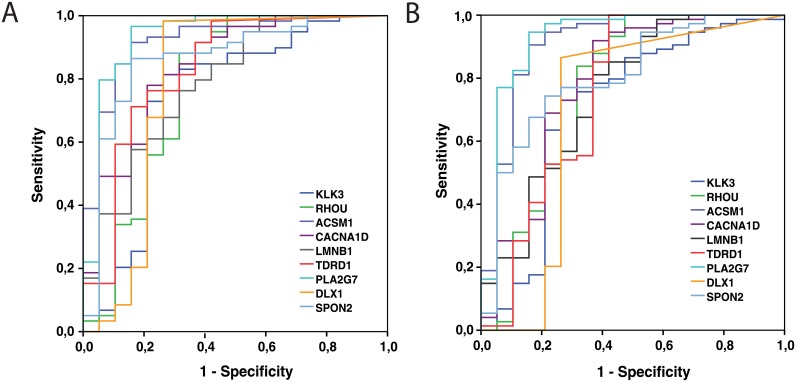
ROC analyses for KLK3 mRNA levels and expression levels of 8 target mRNAs in cases classified as positive or negative for PCa. To simplify the analysis, patients with two cancerous/benign samples were represented by a single value of mRNA expression of each gene. A. RP-PCa samples were considered as positive samples and compared against all CP samples (defined as negative). High sensitivity and specificity was observed for all eight biomarkers. B. RP-Be samples were considered as positive samples and compared against all CP samples (defined as negative).

To assess the sensitivity and specificity of the candidate biomarkers, ROC analysis was used and the area under the curve (AUC) measured for each prospective biomarker (Table 5 and Fig 4). RP-PCa samples were considered as positive samples by definition. Even when utilizing only one of the two available samples for each patient, and comparing these samples against all CP samples (defined as negative by default), particularly high sensitivity and specificity was observed for all eight biomarkers. The AUCs calculated varied between 0.74 and 0.93, indicating 93% specificity in the best cases (Table 5). To examine the importance of the cancer field effect and to implement the predictive power of these genes for very early stages of PCa progression, all RP-B samples were considered by default as positive for cancer (one sample for each patient for which two benign samples were available). When comparing histologically benign samples from cancer patients against all CP samples, which were defined as negative, particularly high AUC values (varying between 0.69 and 0.92) were observed for all of the eight genes.

**Table 5:** AUCs (Area under the Curve) calculated for each of the eight biomarkers and KLK3 for comparison. The table presents the AUC values for each gene in the ROC analyses.

Target mRNA	RP-PCa (only one sample per patient n = 59) Vs. CP (n = 19)	RP-Be (only one sample per patient n = 74) Vs. CP (n = 19)
*KLK3*	0.740	0.709
*RHOU*	0.773	0.761
*ACSM1*	0.921	0.910
*CACNA1D*	0.839	0.784
*LMNB1*	0.789	0.751
*TDRD1*	0.830	0.747
*PLA2G7*	0.935	0.927
*DLX1*	0.783	0.698
*SPON2*	0.863	0.804

### Functional gene knock-down studies by siRNA transfection

Systematic knock-down of all eight marker genes could be successfully performed in VCaP and PC3 cells. SiRNA transfection in LNCaP cells turned out to be highly variable and inconsistent, and not included here. For all cell lines, we used Hiperfect as the most effective transfection reagent, showing consistently very low cytotoxicity, and allowing comparably high concentrations of siRNAs to be used. When testing the knock-down efficacy of different specific siRNAs for different gene, qRT-PCR measurements revealed maximum knock-down levels between 69% to 95% in PC-3 and VCaP cells (Table 6). For seven of the eight genes, a single siRNA was shown to be most effective transfection and superior to pooled siRNA. Transfection of VCaP cells by siRNAs was more difficult compared to PC3, but could be increased after further optimization of the standard transfection protocol.

**Table 6:** Knock-down efficacy as confirmed by qRT-PCR after transfection of PC3 cells with corresponding siRNAs.

Target gene	Knock-down level
*ACSM1*	69%
*CACNA1D*	86%
*DLX1*	82%
*LMNB1*	80%
*PLA2G7*	95%
*RHOU*	91%
*SPON2*	not expressed in PC3 cell line
*TDRD1*	88%

### Knock-down of candidate genes in VCaP organoid cultures

Since VCaP cells did not show striking morphometric changes after siRNA knock-down in either 2D or 3D conditions, we measured the effect of siRNAs on organoid growth and proliferation in round bottom plates indirectly, using the CellTiter-Glo assay. These results are summarized in Table 7: Organoid proliferation as measured by the CellTiter-Glo assay was affected significantly only by knock-down of *DLX1*, *PLA2G7*, and *RHOU*; the strongest effects were seen with AllStar control siRNAs. In addition, the impact of siRNA silencing on apoptosis in VCaP organoids was assessed by the NucView caspase 3/7 assay. Furthermore, the number of dead and dying cells inside VCaP organoids was measured by incorporation of ethidium homodimer, and numbers were quantified by image analyses. The results for VCaP organoids in 3D culture are summarized in Table 8. The most prominent effects on cell death in VCaP organoid are exerted by knock-down of *RHOU* and *CACNA1D*. Knock-down of *DLX1* and TDRD1 also result in measurable, but smaller induction of apoptosis and cell death.

**Table 7:** Growth inhibition of VCaP cells by siRNA knock-down in 2D culture, as measured by the CellTiterGlo assay (2000 cells/well; 384-well microtiter plate).

Untreated	*PLA2G7*	*DLX1*	*RHOU*	*CACNA1D*	*TDRD1*	*LMNB1*	*SPON2*	*ACSM1*	ALLstar
100.0%	79.2%	82.4%	91.0%	103.6%	102.3%	107.3%	95.7%	102.1%	53.1%

**Table 8:** Impact of gene silencing on spheroid size, relative degree of apoptosis, and number of dead cells in VCaP spheroids.

Sample	Apoptosis mean	Apoptosis total signal	EthD mean	EthD total signal	Spheroid area total sum in wells
UNTREATED	100%	100%	100%	100%	100%
SCRAMBLED	99%	88%	111%	95%	89%
*DLX1*	101%	102%	136%	136%	100%
*RHOU*	108%	146%	248%	322%	134%
*SPON2*	122%	107%	120%	103%	87%
*TDRD1*	133%	99%	220%	158%	78%
*LMNB1*	114%	153%	184%	243%	133%
*CACNA1D*	115%	142%	187%	233%	125%
*PLA2G7*	115%	102%	106%	92%	89%
*ACSM1*	108%	102%	117%	117%	98%
ALLSTAR	124%	94%	356%	264%	75%

### Knock-down of *RHOU* and *PLA2G7* decreases the invasion and motility of PC-3 cells

**Fig 5:**
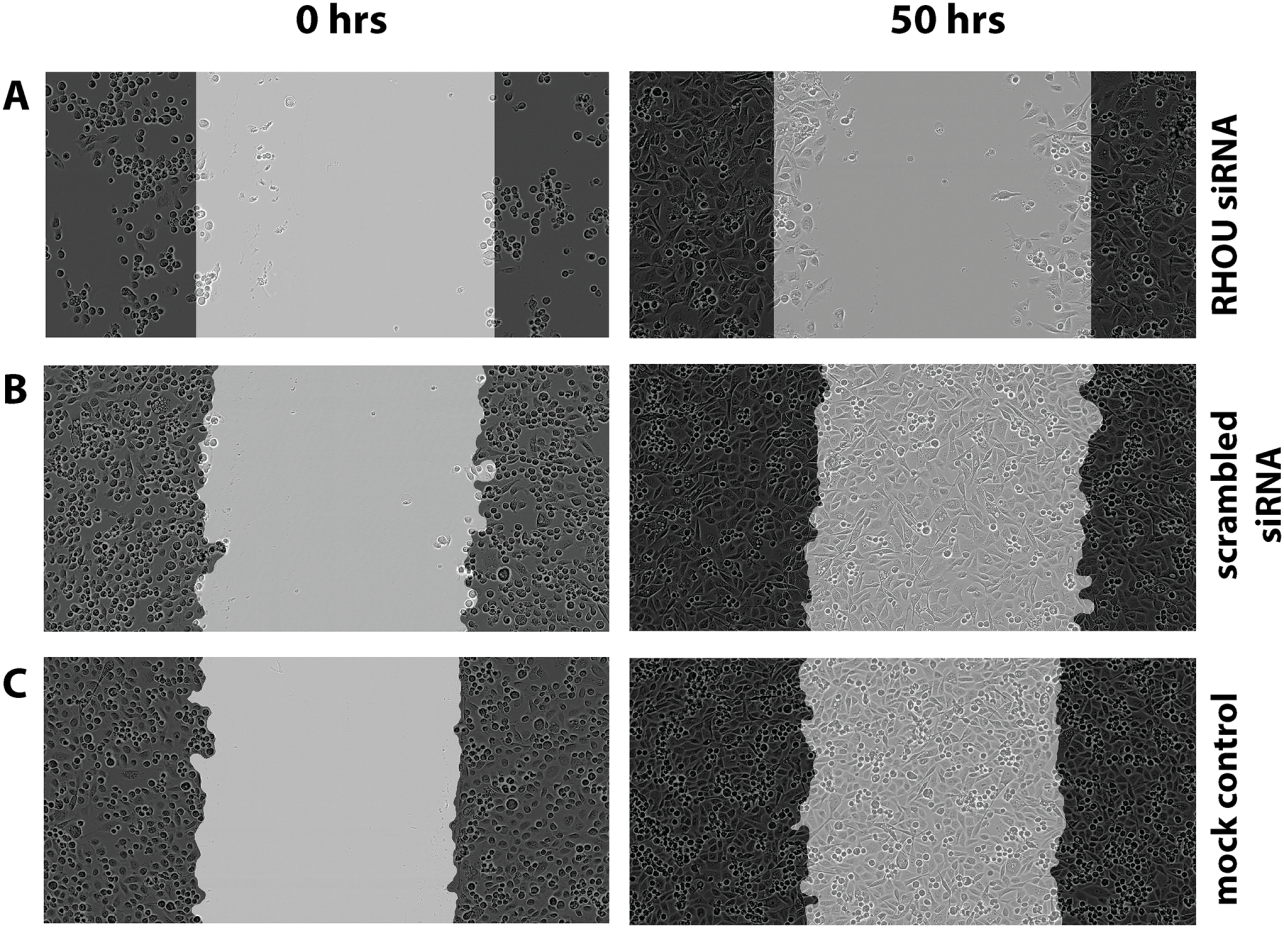
Wound healing assay with PC-3 cells following a 72-hour siRNA transfection. The wound healing results of PC-3 cells treated with RHOU and scrambled siRNA vs. untreated PC-3 cells (mock transfection). Representative images taken 50 h after scratching/wound healing.

**Fig 6:**
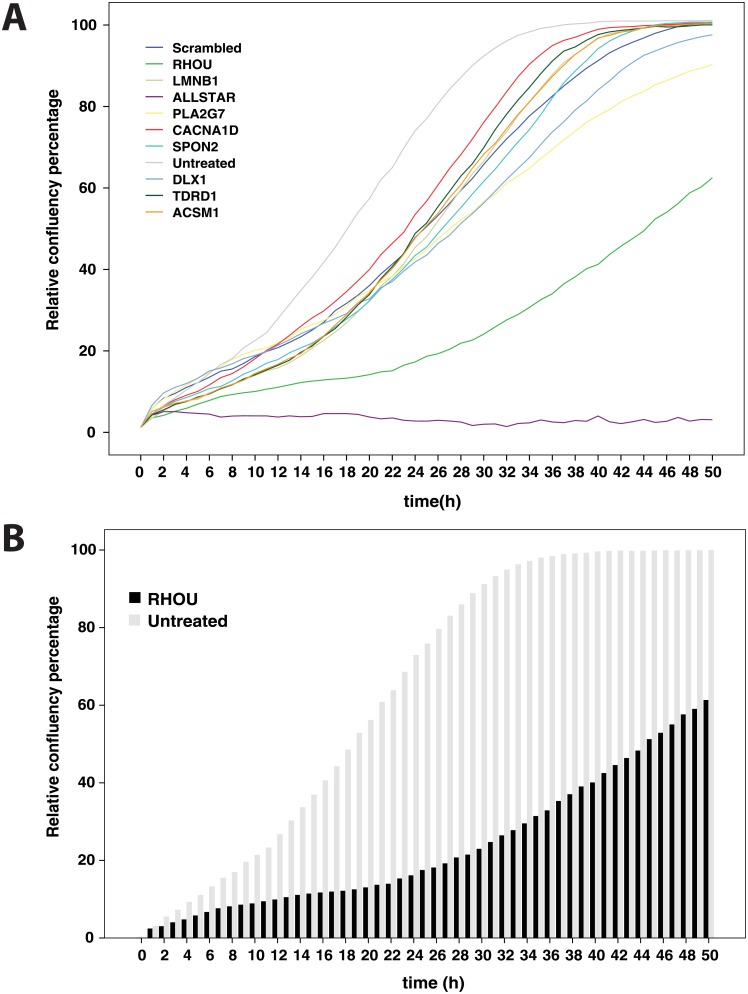
Wound healing curves of untreated (scrambled controls) and specific gene silencing experiments by siRNA, transfected into PC-3 cells. A. Wound healing curves of untreated PC-3 cells and PC-3 cells after siRNA mediated knock-down of all eight candidate genes. AllStar control siRNA induces programmed cell death and was used here as a control for efficacy of siRNA transfection. B. Wound healing/cell migration for RHOU-silenced PC-3 cells, compared to scrambled control.

The effect of silencing the expression of different genes on cell migration and motility was evaluated by wound healing experiments. Under 2D cell culture conditions, the comparison of cells transfected with scrambled siRNA or untreated (mock transfected) cells revealed only slight difference in cell motility and migration for seven of the eight genes under investigation. However, PC-3 cells with knock-down of *RHOU* expression showed strong, reproducible combined effects on both proliferation and cell motility or migration (Fig 5), in comparison with untreated cells and those transfected with scrambled control siRNAs (Fig 6). In addition, silencing of *PLA2G7* resulted in only small, but reproducible effects on cell motility. VCaP cells lack invasive properties and were not used in these assays.

### Effects of gene silencing on multicellular 3D morphology and tumor growth

Functional validation and siRNA knock-down experiments were also performed in 3D organotypic culture, focusing on PC3 cells because VCaP organoids had to be grown in non-adherent suspension cultures and did not show prominent morphometric differences after siRNA silencing. With PC3 cells however, organoids readily formed in 3D culture conditions, and we tested morphometric changes in organoid growth and architecture, combined with assessing the growth rate cell death in 3D culture conditions. Results of siRNA knock-down in PC3 organoids, grown in 3D organotypic culture, were significantly more prominent and easier to quantitatively measure, compared to PC3. For this reason, we have focused mainly on PC3 organoids.

**Fig 7:**
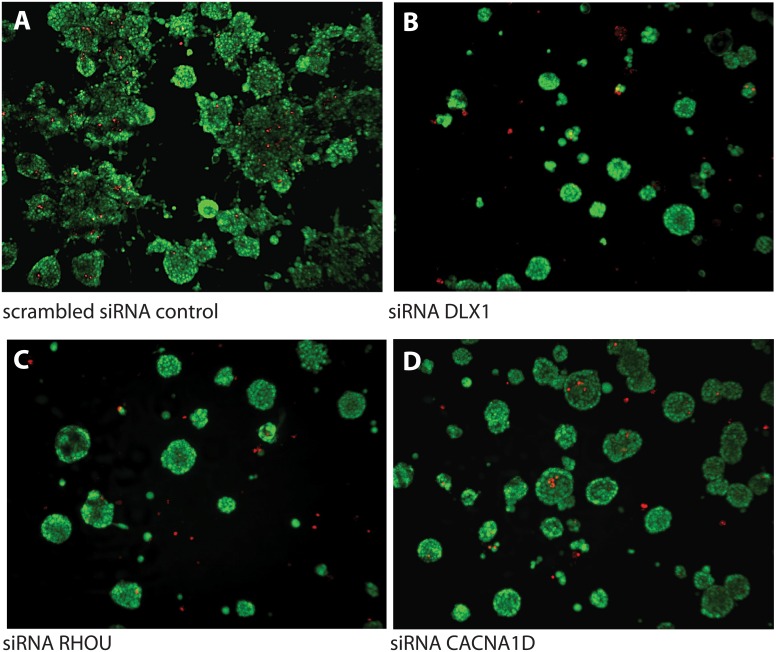
Representative confocal images 3D organoids, formed by PC3 cells embedded in Matrigel. **A**. Control cells were treated with non-functional scrambled siRNA; or with only transfection agent alone. These organoids show multiple invasive processes, typically involving chains of cells (collective invasion pattern). **B**. Silencing of DLX1 results in prominent growth inhibition and formation of small, round, dense and poorly proliferative organoids, but do not show any invasive properties. **C**. Similarly, silencing of the RHOU gene and **D**. of the CACNA1D calcium channel result in round organoids devoid of any invasive processes.

Knock-down of *DLX1* in PC3 cells resulted in the most significant, phenotypic effects upon gene silencing. In comparison to untreated PC3 cells (Fig 7A), RNAi for *DLX1* causes characteristic stunted organoid growth, and resulted in small organoids with high cell density (Fig 7B). This coincides with a complete block of invasive properties. Interestingly, the number of apoptotic cells (red spots in Fig 7A and 7B) is not significantly increased, indicating that loss of *DLX1* functions does not trigger programmed cell death, but may primarily affect cell cycle progression instead.

**Fig 8:**
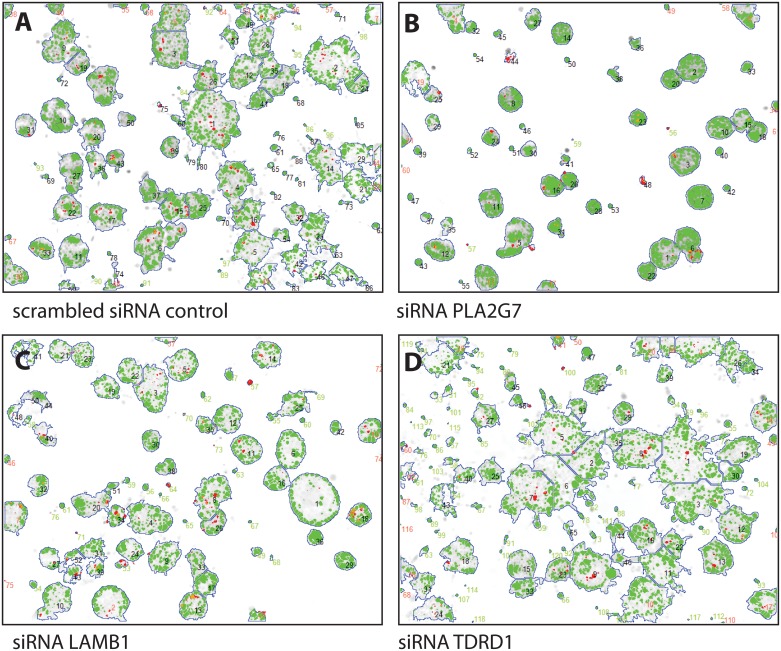
Images of 3D organotypic cell cultures of PC3 cells, embed in Matrigel, after segmentation and subsequent image analysis using the AMIDA software package. A. Untreated cells form large organoids with overt invasive processes. B. Silencing of PLA2G7 and C. laminin beta 1 (LMNB1) result in well-rounded, poorly invasive organoids, with higher cell density. D. In contrast, silencing of the TDRD1 gene results in significant induction of invasive properties, further loss of the structural organization or maturation of organoids, and decreased cell density.

In comparison, knock-down of the *RHOU* (Fig 7C) and *CACNA1D* genes (Fig 7D) resulted in larger, less strongly growth-inhibited organoids compared to *DLX1*, which were significantly smaller than those observed in scrambled siRNA controls. Also *RHOU* and *CACNA1D* silencing effectively inhibited invasive properties observed in untreated PC3 cultures, however with smaller, less significant cytotoxic and growth-inhibitory effects. Silencing of the genes *PLA2G7* (Fig 8B) and *LAMB1* (Fig 8C) resulted in a similar, more or less specific blocking of the invasive properties of PC-3 organoids observed in an organotypic 3D microenvironment. Also here, silencing of *PLA2G7* may be more cytotoxic compared to *LAMB1*, indicated by smaller and denser organoid structures. In contrast, silencing of the *TDRD1* gene (Fig 8D) resulted in opposing effects, leading to more prominent invasive features and generally larger organoids, compared to untreated controls.

**Fig 9:**
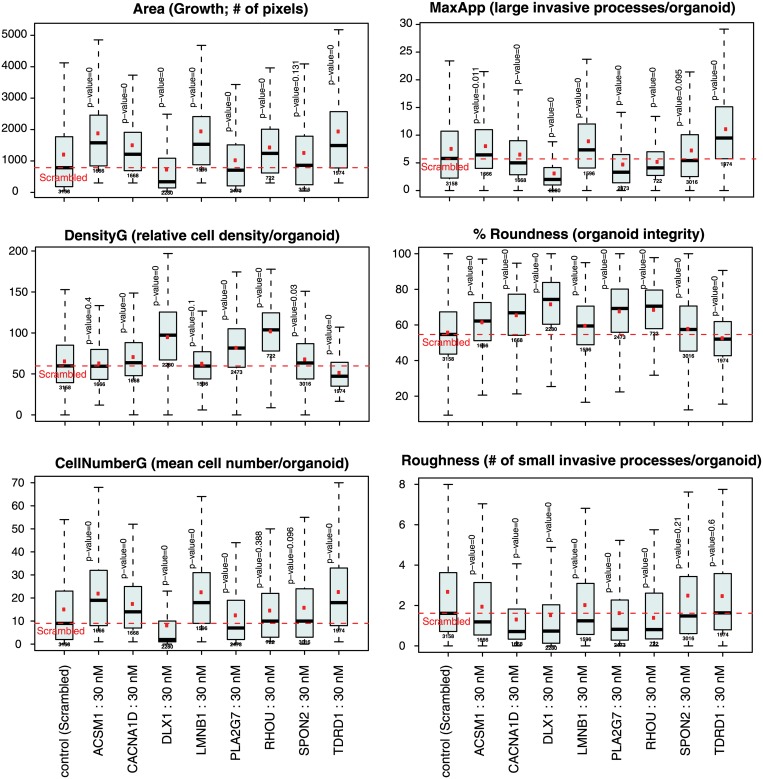
Quantitative assessment of phenotypic changes as the result of siRNA silencing in organotypic 3D cultures.

The impact of siRNA gene silencing on growth and invasive properties in 3D cultures could also be quantitatively analyzed, based on the AMIDA automated image analysis software that measures various morphometric features. These results are summarized in Fig 9. Knock-down of *DLX1* is the only treatment that results in a very significant reduction of organoid size (Area). As described above, this is characteristically concomitant with increased density (DensityG) and a reduced average number of cells inside the organoids (CellNumberG; all shown on left panel of Fig 9).

In contrast, siRNA-mediated silencing or knock-down of the genes *RHOU*, *PLA2G7*, *CACNA1D* and *LAMB1* does not result in smaller organoid size (Area and CellNumberG unchanged or even slightly increased), although their structural density can be increased (DensityG). Nevertheless, silencing of both *RHOU* and *PLA2G7* cause a very significant reduction of macroscopic invasive features (reduced MaxApp and Roughness measures), combined with strongly increased Roundness.

The situation for *LAMB1* and *CACNA1D* silencing are less obvious and clear. Nevertheless, also here the Roughness measure (indicating small, single-cell or subcellular structures invading from the organoid into the microenvironment) is significantly reduced; which coincides with increased Roundness (indicating a more pronounced maturation or differentiation of the organoids).

Silencing of *TDRD1* results in observations that are largely opposing to interfering with any of the other seven candidate genes. *TDRD1* knock-down causes the PC3 organoids to significantly increase in size (Area +), but these simultaneously are less dense (DensityG) although they contain more cells (CellNumberG) and also show many more invasive structures (MaxApp), and are slightly less rounded (Roundness). This indicates that *TDRD1* gene functions may be related to regulation of cell-cell interactions, maturation, differentiation, and integrity of three-dimensional tissue-like structures.

## Discussion

Although serum PSA is widely used as a good indicator for early detection of PCa and tumor load, but has only poor prognostic value in clinical practice, and does not support timely therapy management and intervention. Increase of serum PSA levels is typically delayed and does not increase with the onset of therapy failures and recurrence of tumors. PSA is therefore not predictive for the development of metastatic CRPC. New biomarker candidates are needed that allow a more timely management of the disease, and may ideally predict emerging relapses or therapy failures prior to the recurrence of large tumor masses, such as local lymph node or distant bone metastases. Due to the pronounced heterogeneity and complex nature of CRPC, it would be most beneficial if existing panels of biomarkers, including PSA and maybe other kallikreins, could be combined with additional, novel biomarkers that are more specific for PCa and the mechanisms of disease progression. In practice, this relates to the identification of biomarkers or marker panels that could clearly distinguish indolent from aggressive cancers; or in contrast indicate early stages of progression including field effects and multifocal cancer initiation [7]. Ideally, these markers may also be functionally correlated with and involved in the progression to CRPC. Such earlier detection of therapy failures would allow more timely adaptations to personalized therapy regimens, more individualized and flexible therapeutic decisions, thus saving lives and prolonging patient survival. Most importantly for National healthcare systems, these might also reduce persistent management problems that relate to the frequent over-detection and overtreatment of PCa. A considerable number of studies used gene expression profiles to identify novel diagnostic and predictive biomarkers, or defined multi-gene signatures that may correlate with certain clinical and pathological parameters [32,33]. However, only few predictive markers identified *in silico* have also been validated simultaneously for clinical and functional or mechanistic relevance. For this purpose, we combined i) a genome-wide bioinformatics data mining approach with ii) direct, clinical biomarker validation (using sensitive quantitative RT-PCR), followed by iii) functional evaluation of these candidate genes in disease-relevant processes, such as cancer cell proliferation, motility and invasion.

*Gene selection and prioritization*: Prioritization of candidate genes was performed based on a spectrum of clinical parameters, all intended to simultaneously increase the clinical and functional relevance of these genes simultaneously. These were as follows: i) high or predominant mRNA expression in prostate tissues, and statistically significant correlation with ii) clinical parameters such as Gleason grades, ii) tumor stage, iii) local or distant tumor invasion; iv) lymph node and extracapsular invasion, v) progression to CRPC and metastasis; vi) failure to respond to anti-androgens and vii) association with poor patient survival. Four of the eight genes had not been previously associated with PCa diagnosis or disease progression (*ACSM1*, *RHOU*, *LMNB1*, and *DLX1*). Our data indicate that *CACNA1D*, *DLX1* and *SPON2* might be most informative for prognostic applications, providing the highest predictive value for clinical decision-making. Independent of our studies, *SPON2* was only recently reported [34,35] as a novel marker for blood and serum based biomarker detection of CRPC. In addition, three genes (*CACNA1D*, *TDRD1*, and *PLA2G7*) had been previously reported as potential ERG-target genes [36,37,38,39], specifically induced by expression of the *TMPRSS2-ERG* fusion transcript found in 40–70% of PCas. In addition, Shaikhibrahim and colleagues reported the overexpression of *TDRD1* as an epigenetics-related gene in poorly and moderately differentiated prostate tumors, compared to normal glands [40]. Overexpression of *PLA2G7* as a member of arachidonic acid and prostaglandin pathway in PCa, specifically in *ERG* positive cancers, has been reported [30,41]. Moreover, a specific association of *PLA2G7* with aggressive forms of PCa, it’s prognostic potential as a biomarker, as well as a novel drug target were suggested [31,42]. Furthermore, *PLA2G7* was selected in this study as a positive control gene for our functional evaluation, based on earlier validation studies [43].

*Clinical Validation*: We used quantitative RT-PCR technologies to validate differential expression between normal prostate, benign tissues, and malignant cancers. For seven of the eight candidate genes (all except *PLA2G7*), no predictive association with progression to metastatic CRPC had been previously reported. Only very recently, two of the studied genes (*DLX1* and *TDRD1*, in combination with *HOXC6*) were independently validated as components of a novel prognostic gene signature [44], based on urine sediment diagnostics. Our data confirm these exciting new findings, and validate their relevance as biomarkers. Our study is also relevant as independent evidence for these studies, since they are based on different experimental methods (qRT-PCR), patient cohorts and primary biopsy materials (cystoprostatectomy and radical prostatectomy). All of our eight candidate genes showed statistically significantly different expression between normal/benign prostate and malignant PCa sample groups, low versus high Gleason grade tumors (*PLA2G7*), PSA relapse versus no relapse (*SPON2*), and low versus high TNM stages (*CACNA1D* and *DLX1*). The relevance of all putative prognostic markers was further confirmed by the ROC analyses, indicating generally high AUC values. In all cases, these exceeded the informative, prognostic value of PSA (AUC = 0.740). The highest AUC values were observed for *PLA2G7* (0.935) and *ACSM1* (0.921), followed by *SPON2* (0.863), *CACNA1D* (0.839), *TDRD1* (0.830), *LMNB1* (0.789), *DLX1* (0.783) and *RHOU* (0.773). In addition, all eight genes are differentially expressed between validated normal prostate biopsies. One of the most consistent and striking observations in our study was that for all eight marker genes analyzed here, cancer-adjacent samples show indication of significant field effects, even if they do not contain any detectable cancer tissue. Comparable AUC values for RP-Be vs. CP samples with RP-PCa vs. CP indicates that even in the histologically benign areas, changes in gene expression closely associated with the presence of prostate cancer, could be detected. The prevalence of field effects in PCa is currently only emerging, but may become very important and informative for diagnostic purposes in the near future. Currently, there are very few mechanistic studies that could explain the molecular nature and origin of these field effects in detail.

*Functional Validation*: For the experimental evaluation of gene functions, we utilized organotypic, 3D model systems that recapitulate key aspects of PCa biology, including local invasion into the extracellular matrix (ECM), and specifically investigated the possible involvement of these candidate genes in cell motility, another hallmark of aggressive PCa cells, studied by wound healing assays. Functional validation was performed in both VCaP and PC3 cells. However, technical difficulties such as low siRNA transfection efficacy and the lack of cancer-associated, invasive or motile properties in 2D and 3D growth conditions in VCaP cells have prompted us to focus mainly on the PC3 cell line. Despite their aggressive potential, PC3 cells retain the capacity to differentiate into functional acini-like structures, in particular when embedded in laminin-rich ECM. Such acini spontaneously transform into overtly invasive structures, thus recapitulating the dynamic progression to metastatic CRPC. Although PC-3 cells do not recapitulate all aspects of PCa biology equally well (e.g. due to the lack of androgen receptor expression), they were considered most suitable for our specific functional validation purposes. The most relevant aspects were related to the dynamic invasive potential, pronounced tumor cell plasticity, and ease of siRNA transfection. Significant levels of mRNA expression for seven of the eight candidate genes (except *SPON2*) were detected in PC-3, and all eight were expressed in VCaP cells. Expression of all genes could be effectively reduced by siRNA, and resulted either in increased cell death and apoptosis, reduced growth (in VCaP organoids), or in measurable phenotypic effects in 3D cultures (PC3 organoids). In VCaP cells, silencing of *RHOU*, *LMNB1*, *CACNA1D* and *TDRD1* increased the number of dead cells detectable by the NucView apoptosis assay, or by incorporation of Ethidium homodimer (Table 8). In addition, *DLX1* and *PLA2G7* also resulted in reduced organoid growth, as measured by the CellTiter-Glo assay (Table 7).

In most cases, gene silencing in PC3 cells resulted only in minor cytotoxicity, reduction of tumor cell growth and proliferation, and none induced apoptosis prominently. The strongest proliferation-blocking effects in PC3 organoids were observed for *DLX1*, followed by *PLA2G7* and *RHOU*. *DLX1* is a homebox domain transcription factor of largely unknown function, presumably regulating the (de-) differentiation and integrity of epithelial tissues. Overexpression of *DLX1* in CD26^+^ prostate cancer cells compared to luminal cells has been shown [45]. *DLX1* was only recently identified as overexpressed in many PCas [46], an observation that was now independently confirmed by the new study of *Leyton et al*, [44] and our own work.

In contrast, *PLA2G7*, *RHOU*, *ACSM1*, *LAMB1* and *CACNA1D* silencing resulted mainly in altered cell motility and invasion into the surrounding ECM, with *PLA2G7* and *RHOU* being the most effective. Block of 3D tumor cell invasion coincided with anti-proliferative effects. The effective reduction of cell-motility, combined with a variable degree of cytotoxicity after siRNA knock-down was independently confirmed for *PLA2G7* and *RHOU* in 2D wound healing assays, but not *ACSM*, *LAMB1* and *CACNA1D*. Both *PLA2G7* and *RHOU* genes might be involved in migration-promoting processes. Their specific functions could be related to altering polymerization or contractile properties of the actin cytoskeleton. These processes are more prominent and relevant for collective invasion in 3D culture, but less important for the different modes of cell motility shown in 2D culture on plastic surfaces (amoeboid motility, associated with the formation of lamellipodia and filopodia). *RHOU* is one of the less well-known members of the Rho-family GTPase. Like other Rho family proteins, *RHOU* might also have complex functions in re-arrangement of the actin cytoskeleton, promoting cell-cell adhesion and migration, vesicle trafficking, or regulation of the cell cycle [47]. The ubiquitous role of Rho GTPases in malignant progression is widely established [48]. *RHOU* is an atypical Rho family member with high homology to CDC42, but contains unique N- and C-terminal extensions. The *RHOU* gene was shown to be more efficient than the related Cdc42 in triggering the formation of filopodia and membrane blebbing. *RHOU* also interacts with Rho-Kinases (ROCK) and other modulators of actin cytoskeleton integrity, like PAK1 and NCKbeta [49]. *RHOU* is also functionally linked to Notch1 signaling in leukaemia, where T-ALL cell migration is stimulated by *RHOU* upregulation, leading to enhanced motility and dissemination of leukaemia cells [50]. *RHOU* was further associated with EGF Receptor (EGFR) signaling. It co-localizes and physically associates with activated EGFR; thus leading to AP1 transcriptional activity and effectively promotes migration in pancreatic cancer cells [51]. Similar effects could promote the aggressive and invasive properties of advanced PCas.

Also silencing of *CACNA1D* resulted in morphologic effects and a block of invasive structures, combined with growth inhibition. *CACNA1D* may simultaneously promote PCa cell growth and proliferation [36], and thus play a generalized, supporting role in castration-resistant cancer progression [37]. As for *TDRD1* and *PLA2G7*, progression-associated overexpression of *CACNA1D* may be largely independent of ERG-status in these cancers, and apparently occurs also in ERG-negative cancers. Silencing of *LMNB1* and *ACSM1* had only relatively weak morphologic and invasion-blocking effects. *ACSM1* has not been previously associated with PCa progression, but was reported as a potential marker for the invasive apocrine subtype of breast cancer [52], a rare subtype that is associated with AR+ status and poor differentiation.

Unfortunately, the *SPON2* gene was not expressed in PC-3 cells, and could therefore not be included in functional validation studies. Nevertheless, overexpression of *SPON2* PCa samples and cell lines have been reported previously [53]. Most importantly, increased levels of of SPON2 protein in serum measured by immunoassay shows better diagnostic performance compared to sarcosine and free-to-total and total PSA levels in serum [34]. Furthermore, elevation of serum SPON2 level in PCa patients and its potential to avoid some of the problems of PSA testing due to higher sensitivity and specificity have been documented before [54]. Also hypomethylation of the *SPON2* gene promoter in PCa samples compared with normal samples was reported [55].

In summary, this study provides promising evidence that bioinformatics data mining combined with clinical biomarker validation by qRT-PCR and functional evaluation of candidate genes in disease-relevant processes can be a highly beneficial means to identify novel biomarkers. We have used here siRNA silencing studies to cover all eight target genes across several cell lines. Additional, more detailed functional studies could make use of more precise, but also more time consuming genome editing technologies such as TALENS or CRISPR/Cas9 technologies. This can be considered for detailed follow-up studies on selected candidates in the near future. The eight novel biomarkers studied here show the potential to become useful biomarkers for PCa diagnosis. They may help to improve the timely management of PCa, and support identification of progression to CRPC.

## Supporting Information

S1 File Ranked differentially expressed gene list derived from MSKCC microarray data, comparison metastasis versus primary prostate cancers. (XLS)

S2 File Ranked differentially expressed gene list derived from MSKCC microarray data, comparison normal prostate versus primary prostate cancers. (XLS)

S3 File IST (in silico transcriptomics) plots and dot plots from MSKCC prostate cancer microarray set for all candidate genes. (PDF)

S1 Table List of oligonucleotides primers used for RT-PCR assays. (DOCX)
