# Correction: Validation of Novel Biomarkers for Prostate Cancer Progression by the Combination of Bioinformatics, Clinical and Functional Studies

**DOI:** 10.1371/journal.pone.0158255

**Published:** 2016-06-21

**Authors:** Saeid Alinezhad, Riina-Minna Väänänen, Jesse Mattsson, Yifeng Li, Terhi Tallgrén, Natalia Tong Ochoa, Anders Bjartell, Malin Åkerfelt, Pekka Taimen, Peter J. Boström, Kim Pettersson, Matthias Nees

The gene LAMB1 appears incorrectly throughout the article. The correct gene name should be LMNB1. In Fig 8, the label for Fig 8c is incorrect. Please see the corrected Fig 8 here.

**Fig 8:**
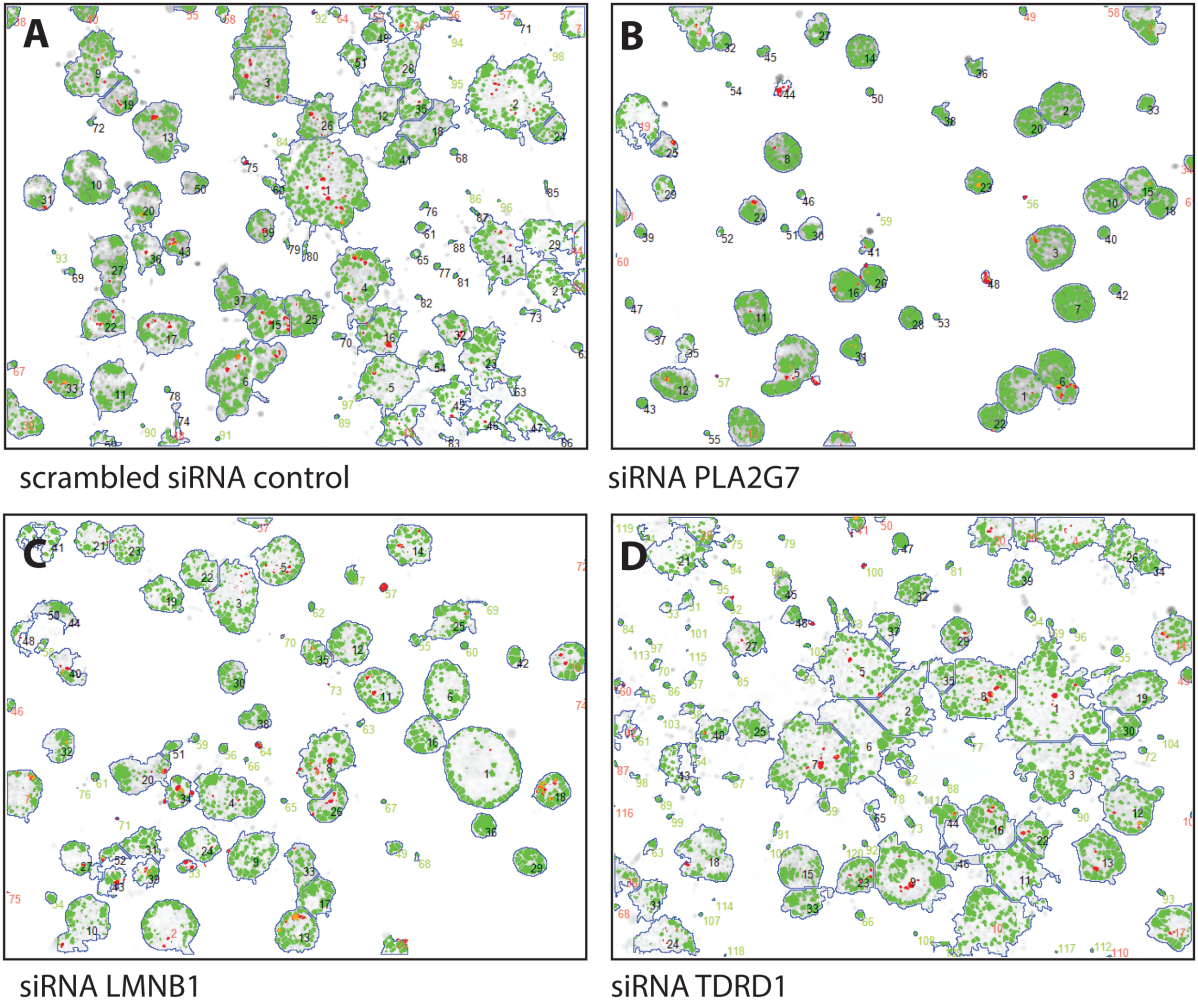
Images of 3D organotypic cell cultures of PC3 cells, embed in Matrigel, after segmentation and subsequent image analysis using the AMIDA software package A. Untreated cells form large organoids with overt invasive processes. B. Silencing of PLA2G7 and C. laminin beta 1 (LMNB1) result in well-rounded, poorly invasive organoids, with higher cell density. D. In contrast, silencing of the TDRD1 gene results in significant induction of invasive properties, further loss of the structural organization or maturation of organoids, and decreased cell density.
