# Supplementary material for: Validation of Novel Biomarkers for Prostate Cancer Progression by the Combination of Bioinformatics, Clinical and Functional Studies
Source: PLoS One. 2016 May 19;11(5):e0155901. doi: 10.1371/journal.pone.0155901 (PMC4873225; doi:10.1371/journal.pone.0155901)
Supplement: S3 File — (PDF) [file pone.0155901.s003.pdf]

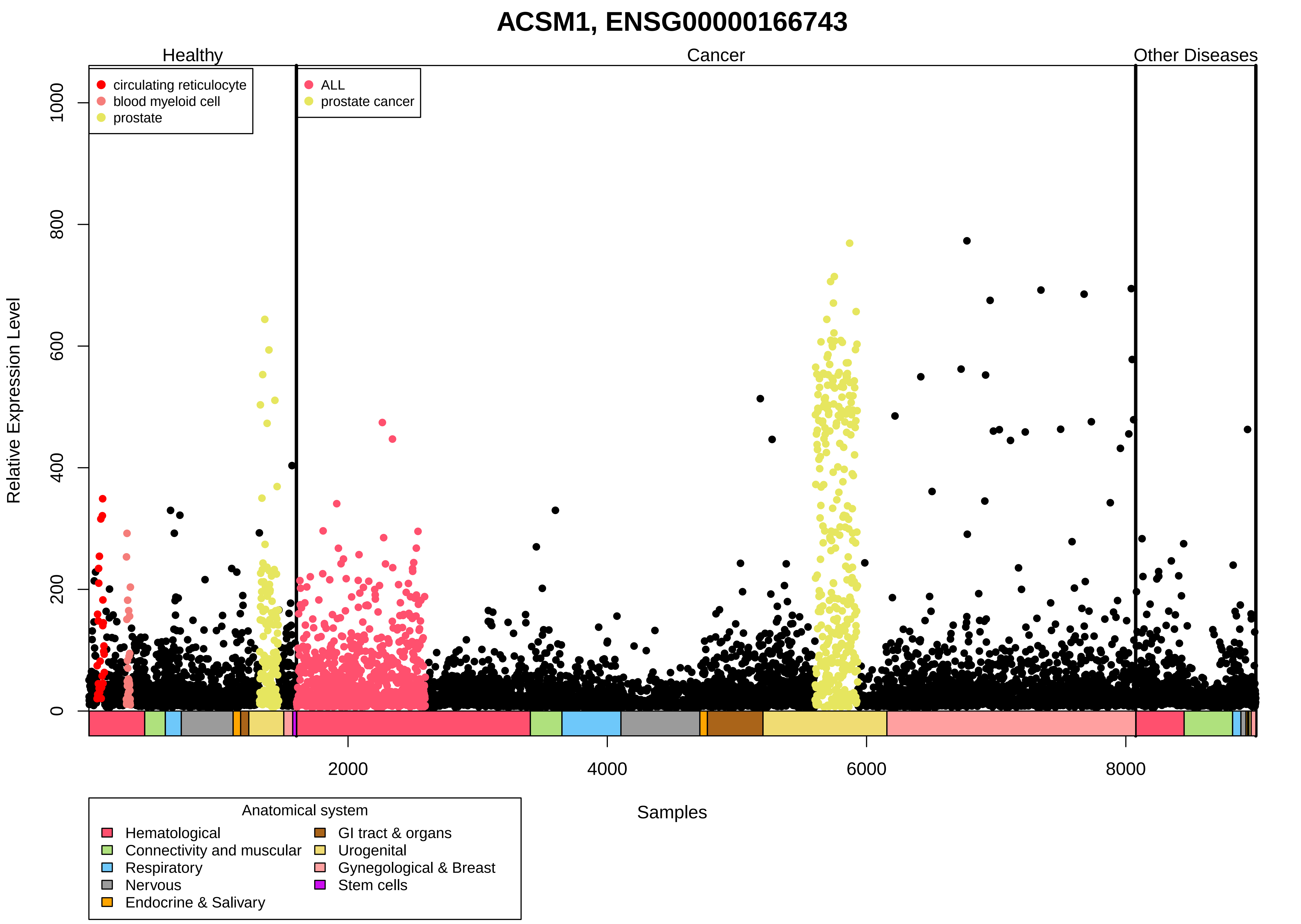
ACSM1, ENSG00000166743
Healthy
Cancer
Other Diseases
circulating reticulocyte
blood myeloid cell
prostate
ALL
prostate cancer
Relative Expression Level
0
200
400
600
800
1000
2000
4000
6000
8000
Samples
Anatomical system
Hematological
Connectivity and muscular
Respiratory
Nervous
Endocrine & Salivary
GI tract & organs
Urogenital
Gynegological & Breast
Stem cells

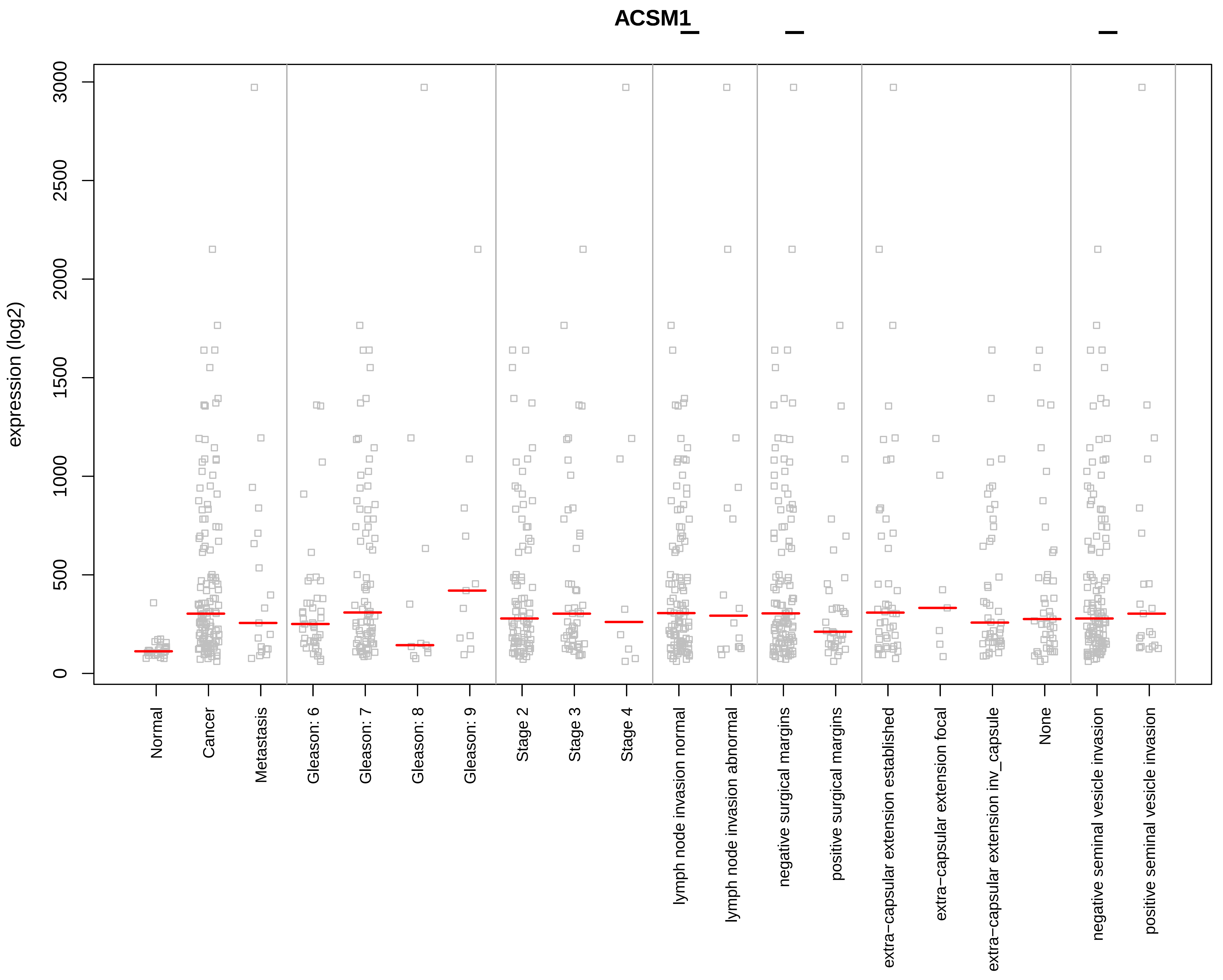

ACSM1
expression (log2)
0
500
1000
1500
2000
2500
3000
Normal
Cancer
Metastasis
Gleason: 6
Gleason: 7
Gleason: 8
Gleason: 9
Stage 2
Stage 3
Stage 4
lymph node invasion normal
lymph node invasion abnormal
negative surgical margins
positive surgical margins
extra−capsular extension established
extra−capsular extension focal
extra−capsular extension inv_capsule
None
negative seminal vesicle invasion
positive seminal vesicle invasion

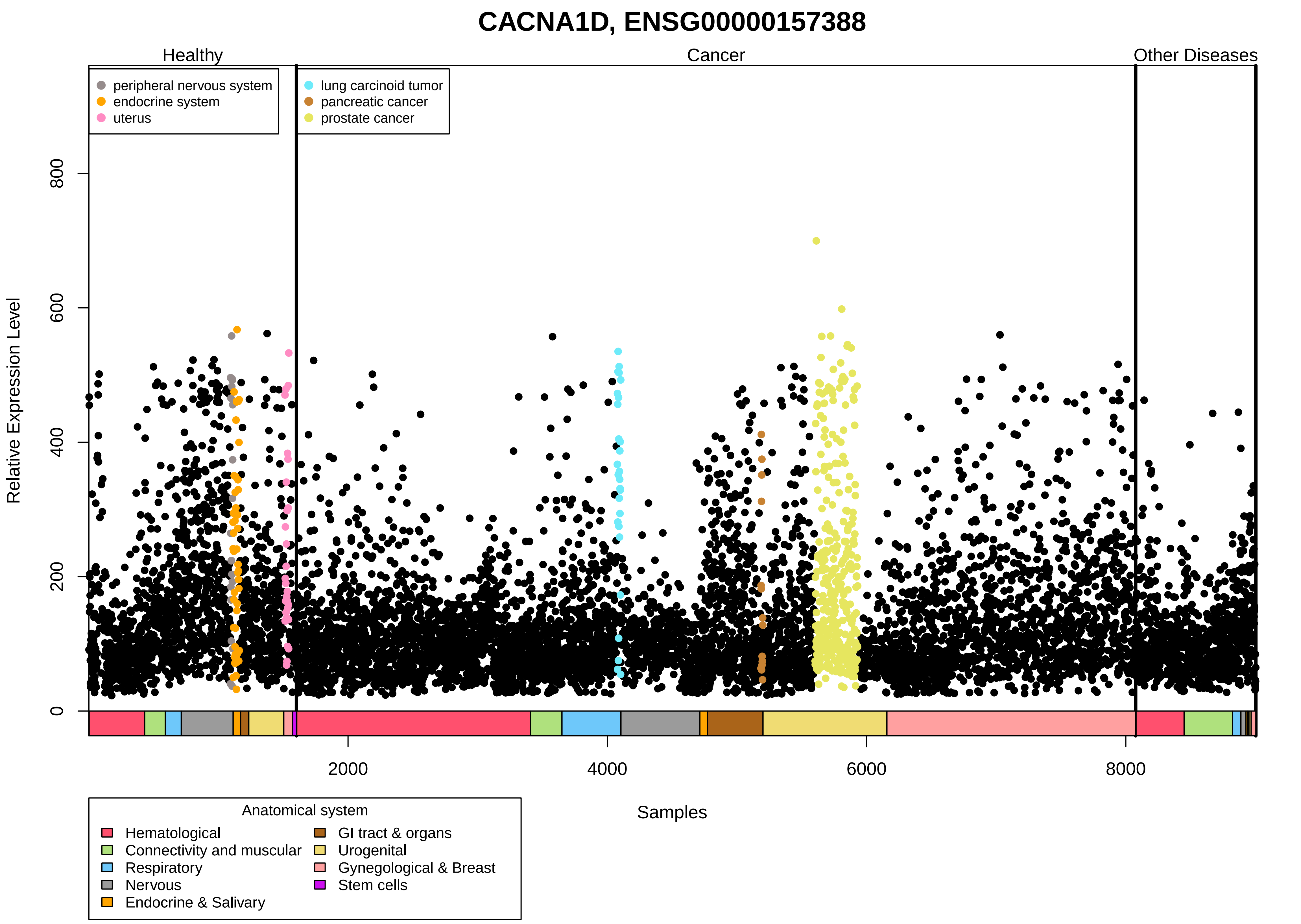

CACNA1D, ENSG00000157388
Healthy
Cancer
Other Diseases
peripheral nervous system
endocrine system
uterus
lung carcinoid tumor
pancreatic cancer
prostate cancer
Relative Expression Level
0
200
400
600
800
2000
4000
6000
8000
Samples
Anatomical system
Hematological
Connectivity and muscular
Respiratory
Nervous
Endocrine & Salivary
GI tract & organs
Urogenital
Gynegological & Breast
Stem cells

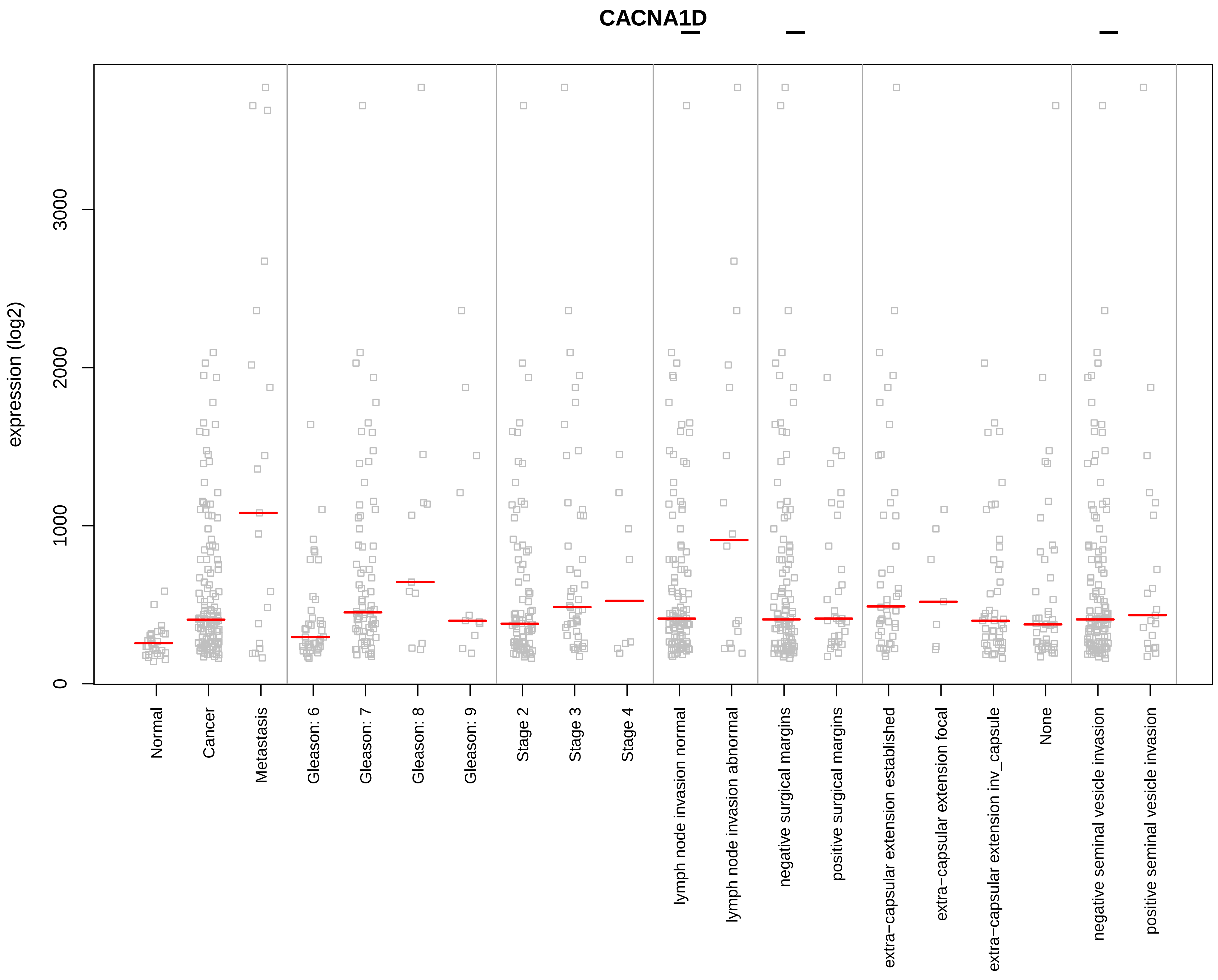

CACNA1D
expression (log2)
0
1000
2000
3000
Normal
Cancer
Metastasis
Gleason: 6
Gleason: 7
Gleason: 8
Gleason: 9
Stage 2
Stage 3
Stage 4
lymph node invasion normal
lymph node invasion abnormal
negative surgical margins
positive surgical margins
extra−capsular extension established
extra−capsular extension focal
extra−capsular extension inv_capsule
None
negative seminal vesicle invasion
positive seminal vesicle invasion

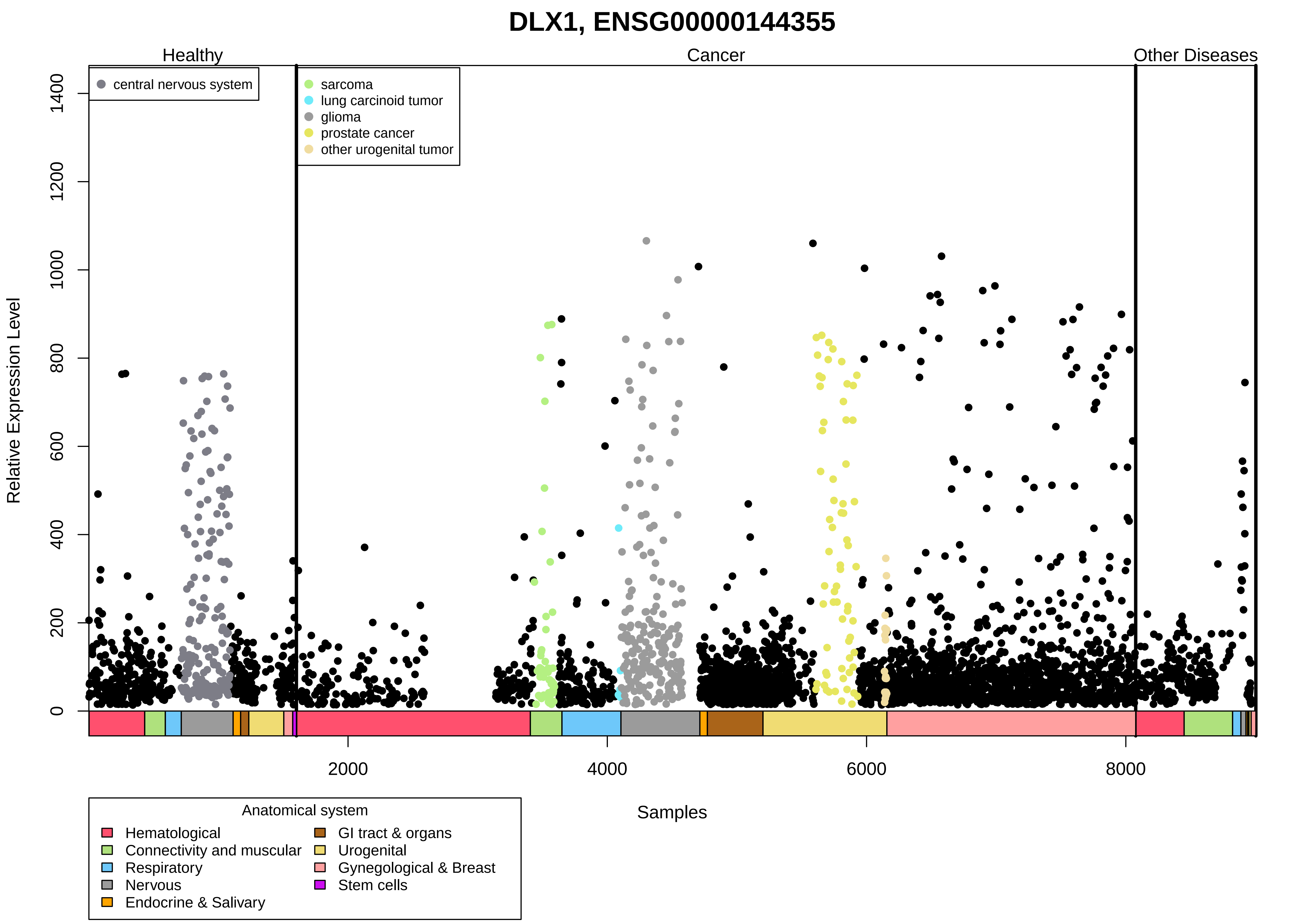

DLX1, ENSG00000144355
Healthy
Cancer
Other Diseases
central nervous system
sarcoma
lung carcinoid tumor
glioma
prostate cancer
other urogenital tumor
Relative Expression Level
0
200
400
600
800
1000
1200
1400
2000
4000
6000
8000
Samples
Anatomical system
Hematological
Connectivity and muscular
Respiratory
Nervous
Endocrine & Salivary
GI tract & organs
Urogenital
Gynegological & Breast
Stem cells

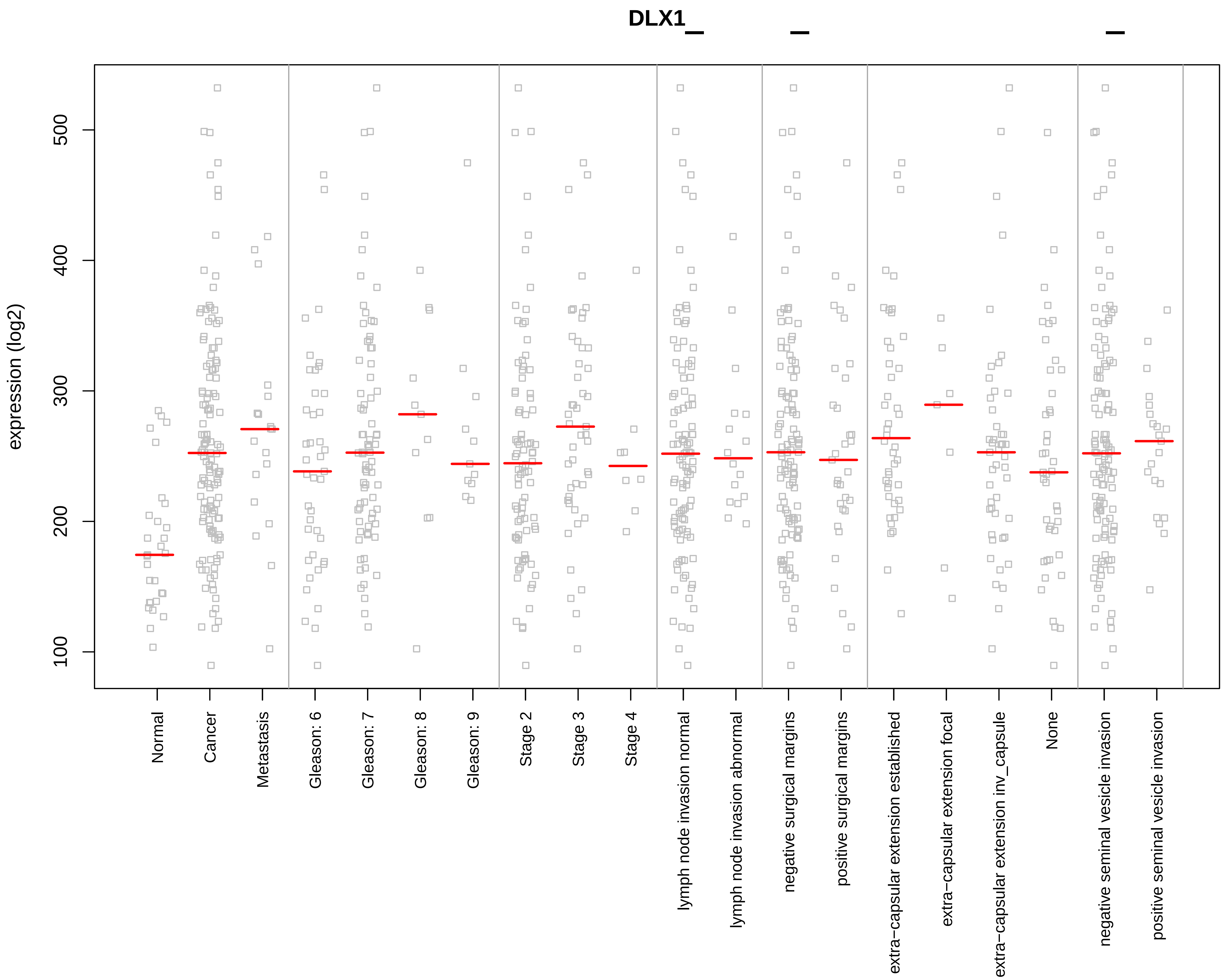
DLX1
expression (log2)
100
200
300
400
500
Normal
Cancer
Metastasis
Gleason: 6
Gleason: 7
Gleason: 8
Gleason: 9
Stage 2
Stage 3
Stage 4
lymph node invasion normal
lymph node invasion abnormal
negative surgical margins
positive surgical margins
extra-capsular extension established
extra-capsular extension focal
extra-capsular extension inv_capsule
None
negative seminal vesicle invasion
positive seminal vesicle invasion

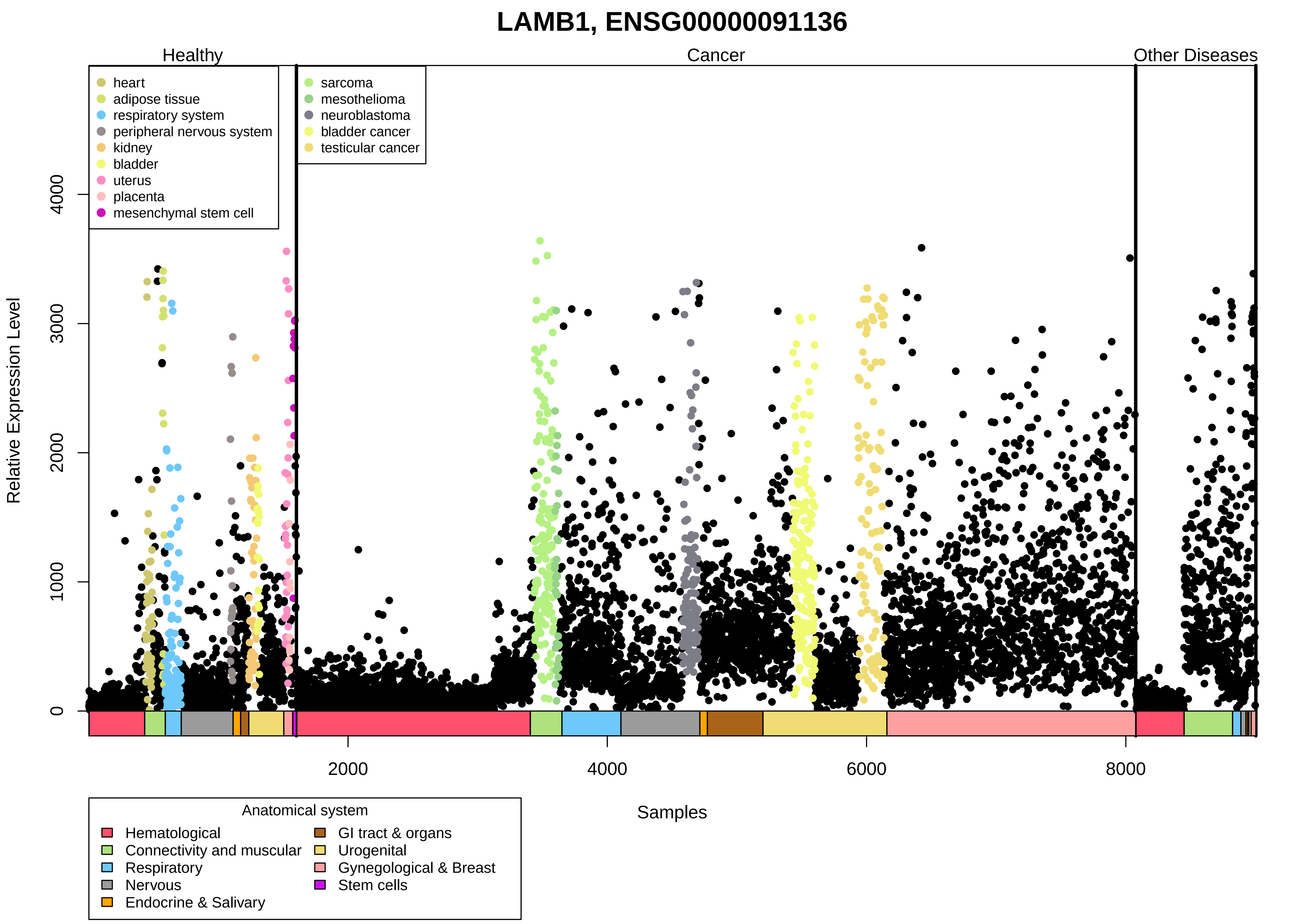

LAMB1, ENSG00000091136
Healthy
Cancer
Other Diseases
heart
adipose tissue
respiratory system
peripheral nervous system
kidney
bladder
uterus
placenta
mesenchymal stem cell
sarcoma
mesothelioma
neuroblastoma
bladder cancer
testicular cancer
Relative Expression Level
0
1000
2000
3000
4000
2000
4000
6000
8000
Samples
Anatomical system
Hematological
Connectivity and muscular
Respiratory
Nervous
Endocrine & Salivary
GI tract & organs
Urogenital
Gynegological & Breast
Stem cells

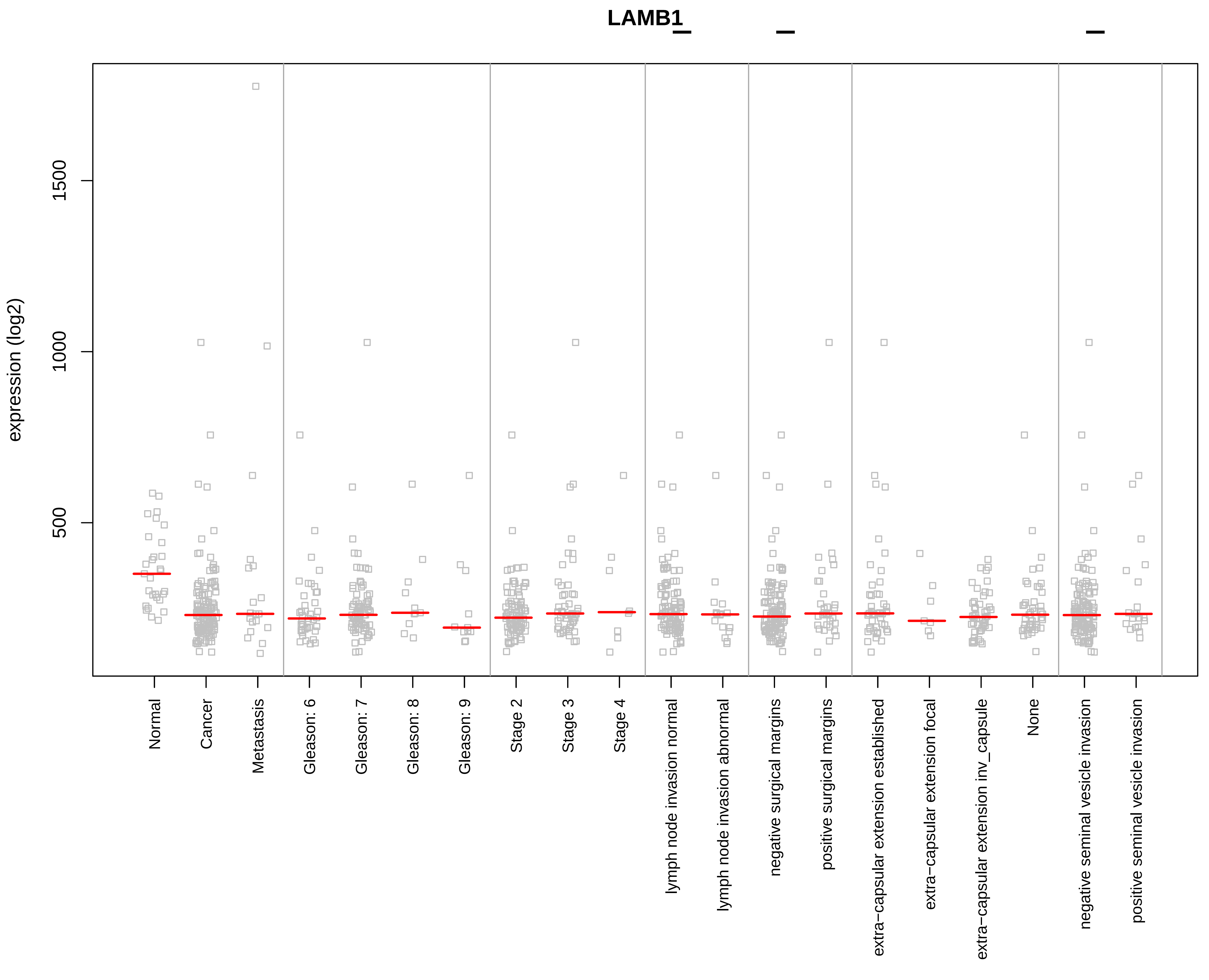

LAMB1
expression (log2)
500
1000
1500
Normal
Cancer
Metastasis
Gleason: 6
Gleason: 7
Gleason: 8
Gleason: 9
Stage 2
Stage 3
Stage 4
lymph node invasion normal
lymph node invasion abnormal
negative surgical margins
positive surgical margins
extra−capsular extension established
extra−capsular extension focal
extra−capsular extension inv_capsule
None
negative seminal vesicle invasion
positive seminal vesicle invasion

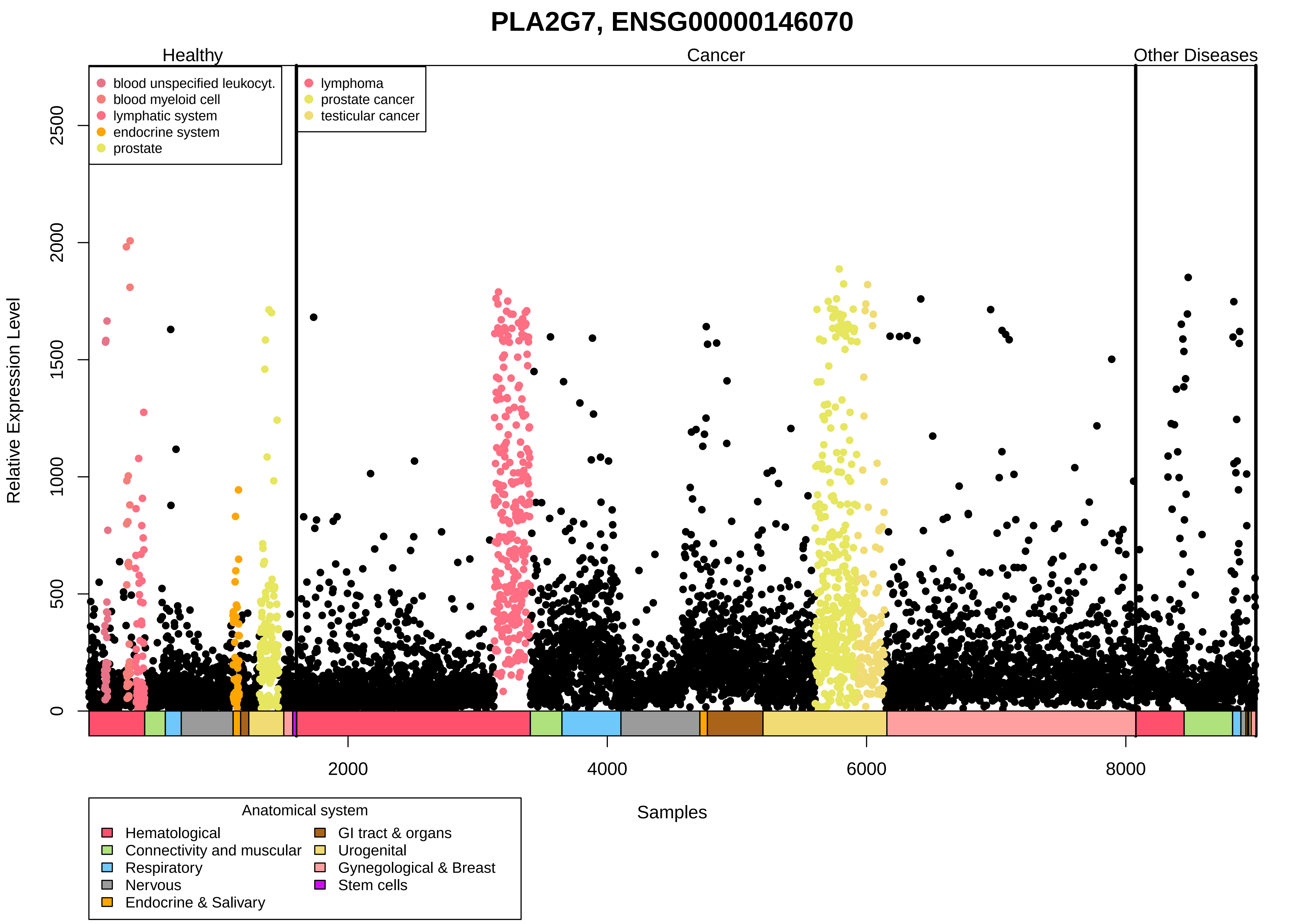
PLA2G7, ENSG00000146070
Healthy
Cancer
Other Diseases
blood unspecified leukocyt.
blood myeloid cell
lymphatic system
endocrine system
prostate
lymphoma
prostate cancer
testicular cancer
Relative Expression Level
0
500
1000
1500
2000
2500
2000
4000
6000
8000
Samples
Anatomical system
Hematological
Connectivity and muscular
Respiratory
Nervous
Endocrine & Salivary
GI tract & organs
Urogenital
Gynegological & Breast
Stem cells

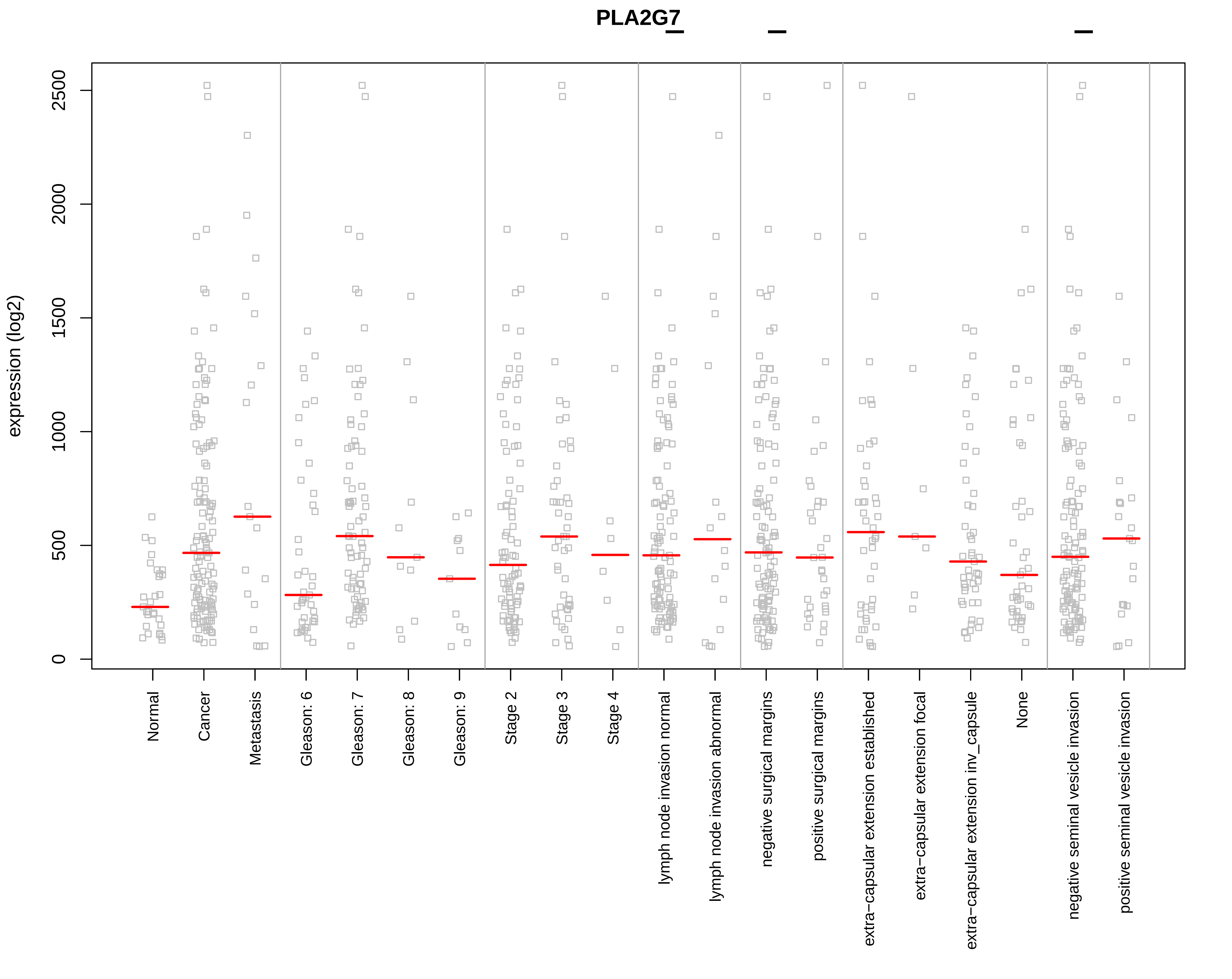

PLA2G7
expression (log2)
0
500
1000
1500
2000
2500
Normal
Cancer
Metastasis
Gleason: 6
Gleason: 7
Gleason: 8
Gleason: 9
Stage 2
Stage 3
Stage 4
lymph node invasion normal
lymph node invasion abnormal
negative surgical margins
positive surgical margins
extra−capsular extension established
extra−capsular extension focal
extra−capsular extension inv_capsule
None
negative seminal vesicle invasion
positive seminal vesicle invasion

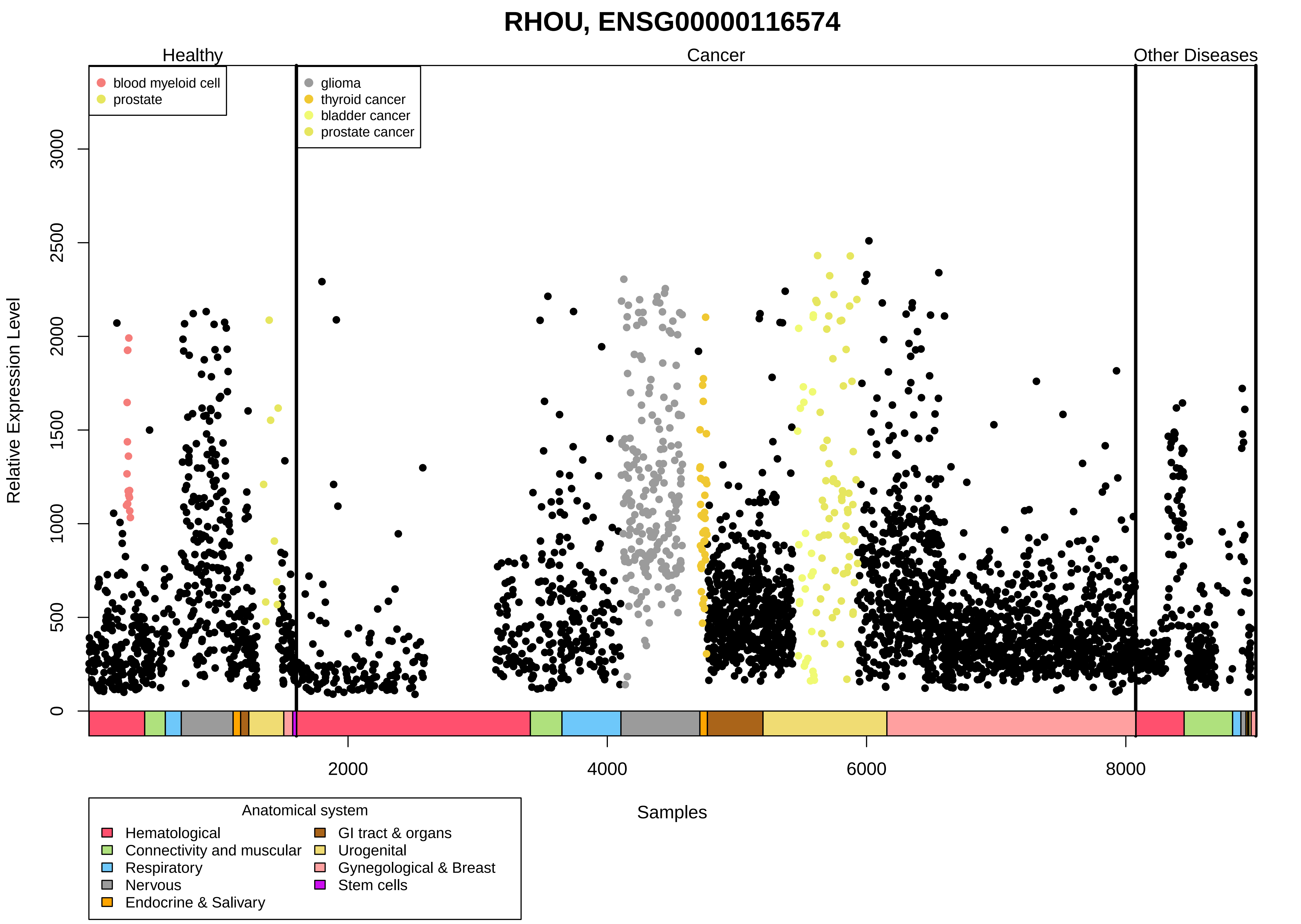

RHOU, ENSG00000116574
Healthy
Cancer
Other Diseases
blood myeloid cell
prostate
glioma
thyroid cancer
bladder cancer
prostate cancer
Relative Expression Level
0
500
1000
1500
2000
2500
3000
2000
4000
6000
8000
Samples
Anatomical system
Hematological
Connectivity and muscular
Respiratory
Nervous
Endocrine & Salivary
GI tract & organs
Urogenital
Gynegological & Breast
Stem cells

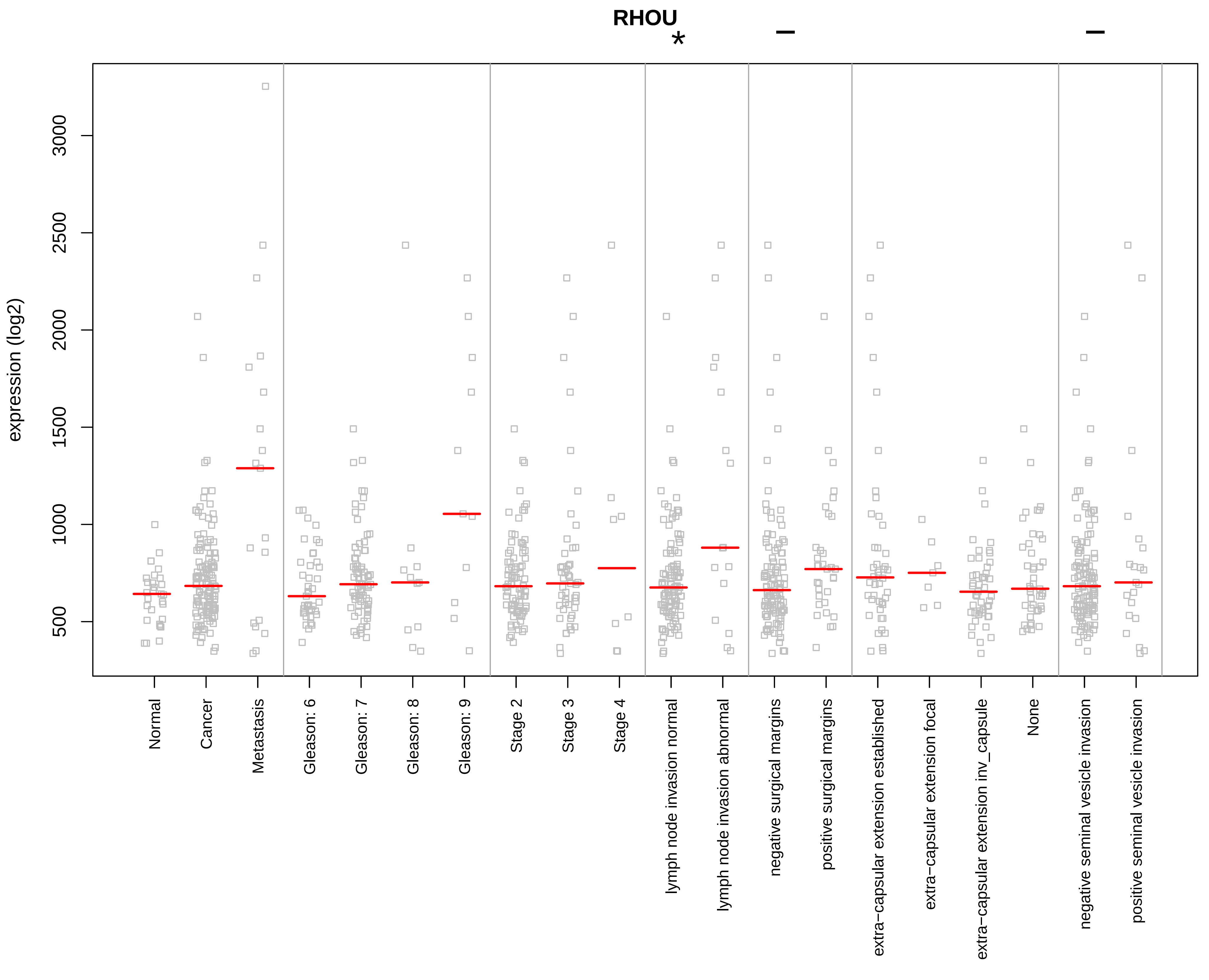

RHOU
*
expression (log2)
500
1000
1500
2000
2500
3000
Normal
Cancer
Metastasis
Gleason: 6
Gleason: 7
Gleason: 8
Gleason: 9
Stage 2
Stage 3
Stage 4
lymph node invasion normal
lymph node invasion abnormal
negative surgical margins
positive surgical margins
extra−capsular extension established
extra−capsular extension focal
extra−capsular extension inv_capsule
None
negative seminal vesicle invasion
positive seminal vesicle invasion

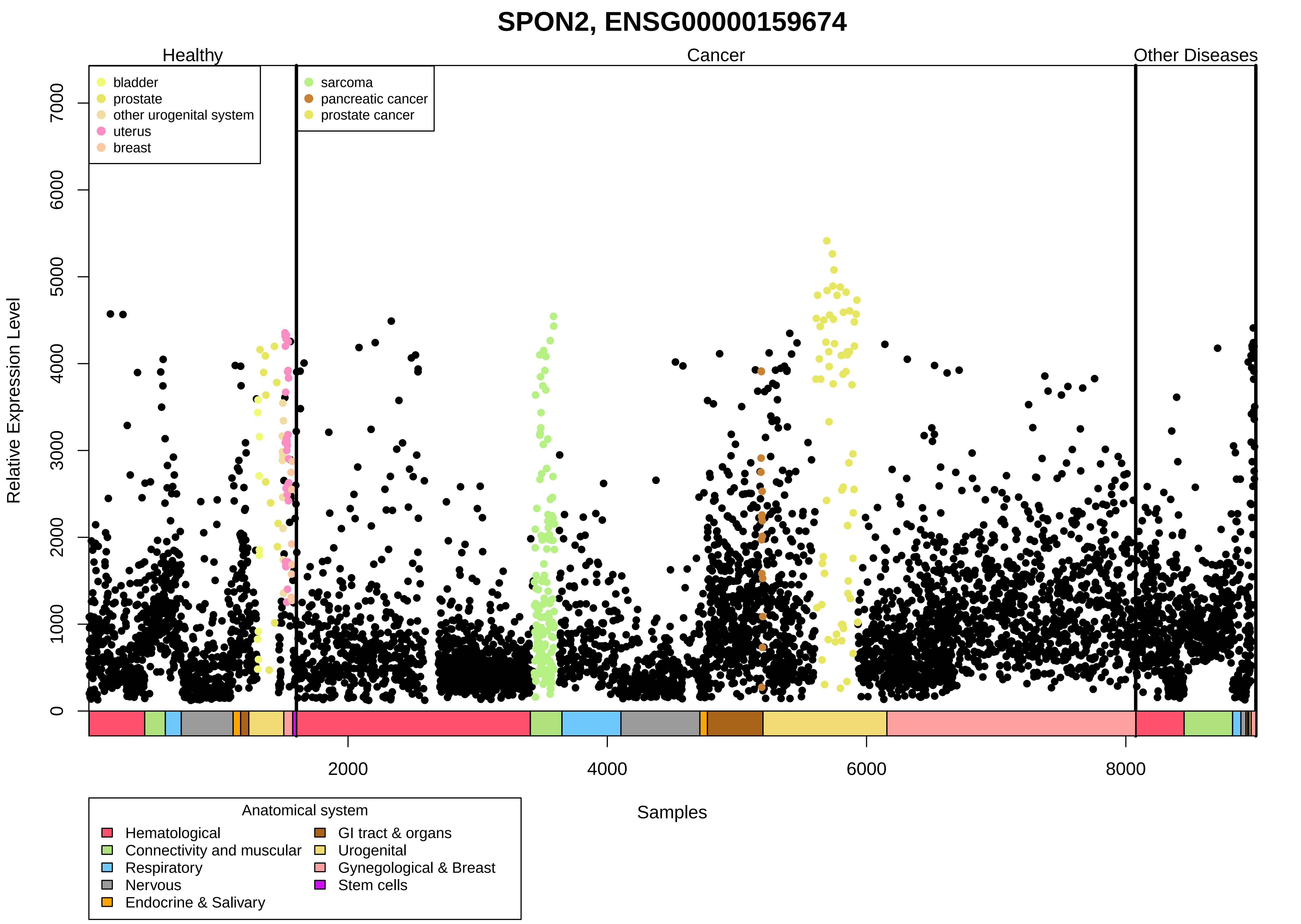

SPON2, ENSG00000159674
Healthy
Cancer
Other Diseases
bladder
prostate
other urogenital system
uterus
breast
sarcoma
pancreatic cancer
prostate cancer
Relative Expression Level
0
1000
2000
3000
4000
5000
6000
7000
2000
4000
6000
8000
Samples
Anatomical system
Hematological
Connectivity and muscular
Respiratory
Nervous
Endocrine & Salivary
GI tract & organs
Urogenital
Gynegological & Breast
Stem cells

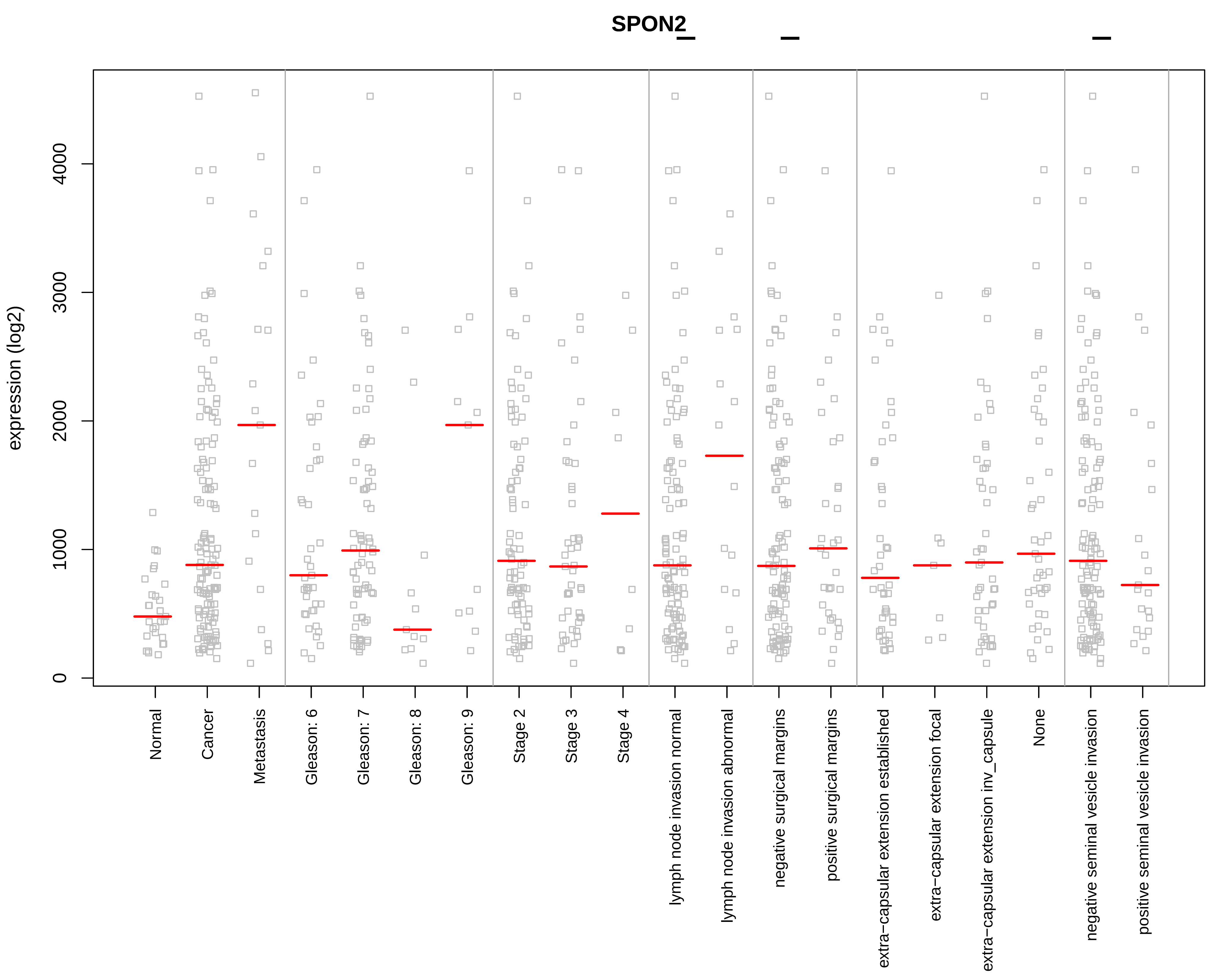
SPON2_
expression (log2)
0
1000
2000
3000
4000
Normal
Cancer
Metastasis
Gleason: 6
Gleason: 7
Gleason: 8
Gleason: 9
Stage 2
Stage 3
Stage 4
lymph node invasion normal
lymph node invasion abnormal
negative surgical margins
positive surgical margins
extra−capsular extension established
extra−capsular extension focal
extra−capsular extension inv_capsule
None
negative seminal vesicle invasion
positive seminal vesicle invasion

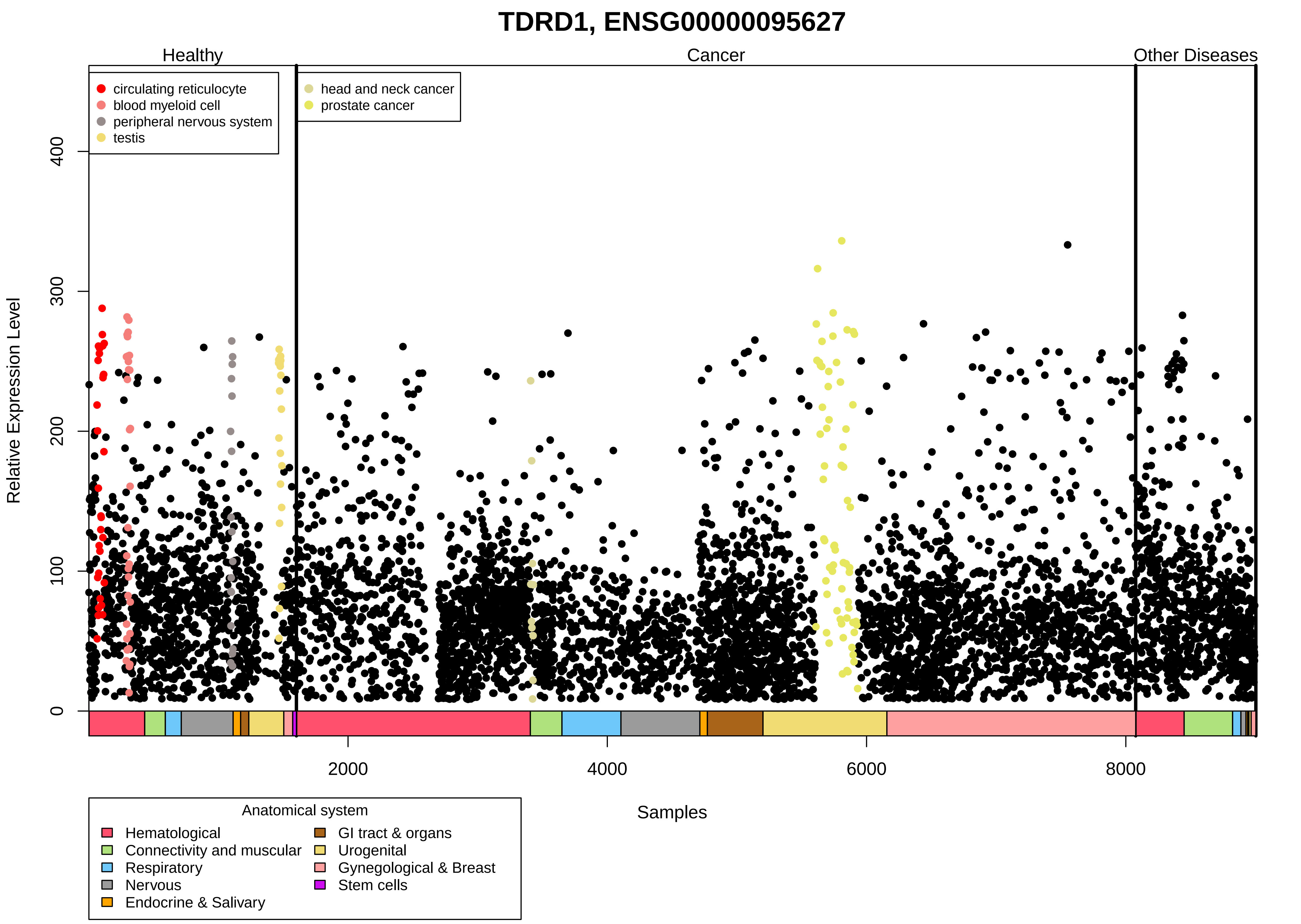

TDRD1, ENSG00000095627
Healthy
Cancer
Other Diseases
circulating reticulocyte
blood myeloid cell
peripheral nervous system
testis
head and neck cancer
prostate cancer
Relative Expression Level
0
100
200
300
400
2000
4000
6000
8000
Samples
Anatomical system
Hematological
Connectivity and muscular
Respiratory
Nervous
Endocrine & Salivary
GI tract & organs
Urogenital
Gynegological & Breast
Stem cells

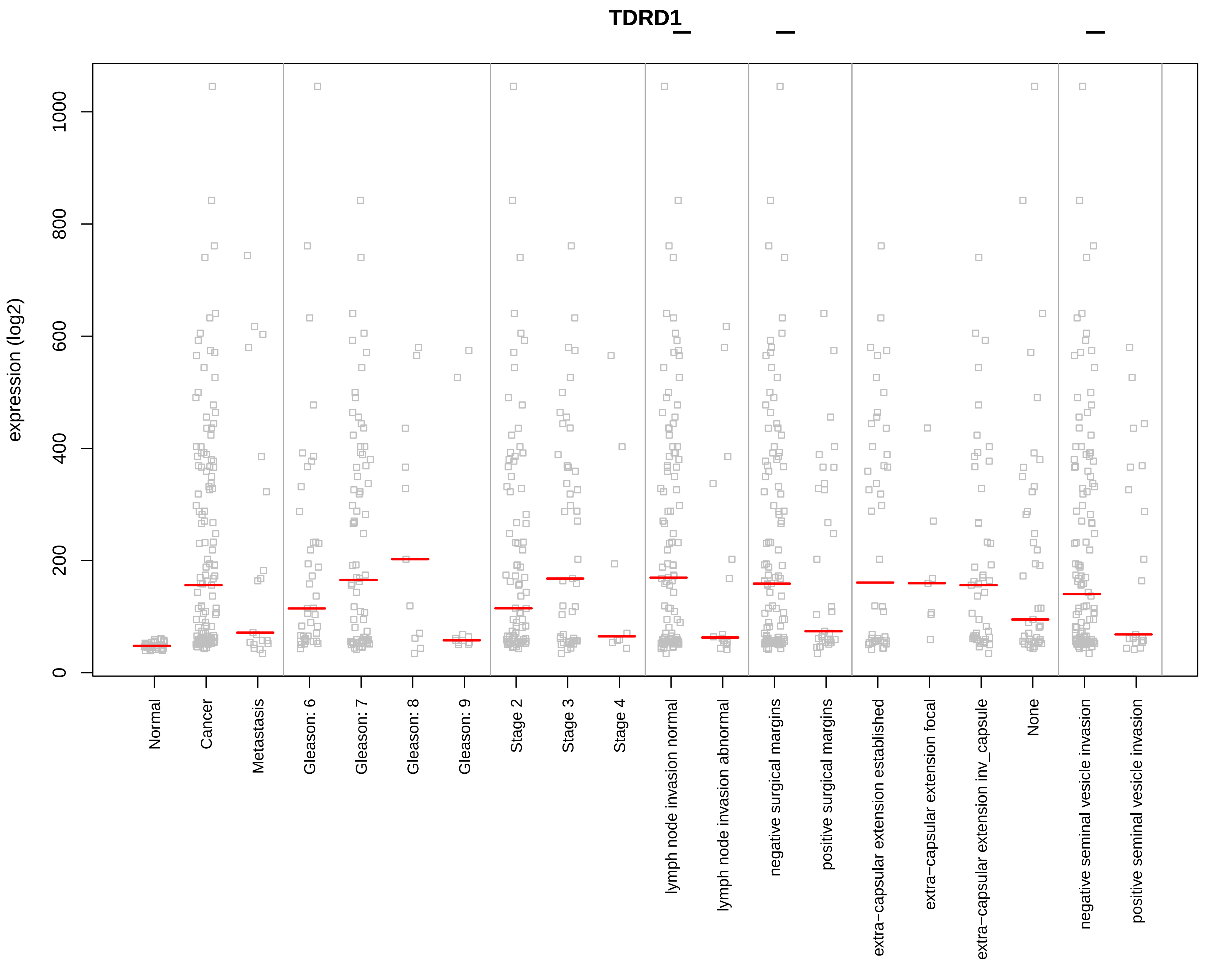
TDRD1
expression (log2)
0
200
400
600
800
1000
Normal
Cancer
Metastasis
Gleason: 6
Gleason: 7
Gleason: 8
Gleason: 9
Stage 2
Stage 3
Stage 4
lymph node invasion normal
lymph node invasion abnormal
negative surgical margins
positive surgical margins
extra−capsular extension established
extra−capsular extension focal
extra−capsular extension inv_capsule
None
negative seminal vesicle invasion
positive seminal vesicle invasion
