# Supplementary material for: Validation of Novel Biomarkers for Prostate Cancer Progression by the Combination of Bioinformatics, Clinical and Functional Studies
Source: PLoS One. 2016 May 19;11(5):e0155901. doi: 10.1371/journal.pone.0155901 (PMC4873225; doi:10.1371/journal.pone.0155901)
Supplement: S1 Table — (DOCX) [file pone.0155901.s004.docx]

Supplemental table 1. List of the oligunucleotides used for RT-PCR assays

| Oligonucleotide | 5' 3' Sequence | Database sequence number | Position in sequence |
| --- | --- | --- | --- |
| *ACSM1*  Forward primer  Reverse primer  Reporter probe  Quencher probe | CCAGGAAGTAGGAAATTACG  GGGTATTTCAACAATGTCTG  TGTCAAACTGTGGCAGATGGTGGATAAAGA  TCTGCCACAGTTTGACA | NM_052956.2  NM_052956.2  NM_052956.2  NM_052956.2 | 813-832  978-997  934-963  947-963 |
| *CACNA1D*  Forward primer  Reverse primer  Reporter probe  Quencher probe | AGGAGTGCCCAGTTTACAAG  TCCTCTTCAGCTACGATATCTG  TTTGTGCATTTTTCCAATAAAAAGTTCCAATCC  TTGGAAAAATGCACAAA | NM_000720.3  NM_000720.3  NM_000720.3  NM_000720.3 | 874-893  1035-1056  983-1015  999-1015 |
| *DLX1*  Forward primer  Reverse primer  Reporter probe  Quencher probe | CAATGGCAAGGGAAAAAAG  GAACCAGATCTTGACCTGAGTC  TCCAGTTTGCAGTTGCAGGCTTTGAAC  TGCAACTGCAAACTGGA | NM_178120.4  NM_178120.4  NM_178120.4  NM_178120.4 | 561-579  705-726  604-630  604-620 |
| *KLK3*  Forward primer  Reverse primer  Reporter probe  Quencher probe | AGCATTGAACCAGAGGAGTTCT  CCCGAGCAGGTGCTTTTG  CCTTCTGAGGGTGAACTTGCGC  AAT CAC CCT CAG AAG G | X05332  X05332  X05332  X05332 | 518 – 539  658 – 675  596 – 617  604 – 617 |
| *LMNB1*  Forward primer  Reverse primer  Reporter probe  Quencher probe | ATGTATGAAGAGGAGATTAACG  CATTCTCAAGTTTGGCATG  TCCAGCTCCTCCTTATACAGCCTCACTT  TGTATAAGGAGGAGCTGGA | NM_005573.3  NM_005573.3  NM_005573.3  NM_005573.3 | 1517-1538  1691-1709  1653-1680  1662-1680 |
| mmPSA  Forward primer  Reverse primer  Reporter probe  Quencher probe | TGAACCAGAGGAGTTCTTGCA  CCCAGAATCACCCGAGCGA  CCTTCTGAGGGTGATTGCGCAC  AATCACCCTCAGAAGG | X05332  X05332  X05332  X05332 | 523 – 543  667 – 685  594 – 601  604 – 617 |
| *PLA2G7*  Forward primer  Reverse primer  Reporter probe  Quencher probe | ATTACAATCAGGGGTTCAGTC  AATCTTTATGAAGTCCTAAATGC  TGCAACTGGCAAAATAATTGGACACATGC  TTATTTTGCCAGTTGCA | NM_005084.3  NM_005084.3  NM_005084.3  NM_005084.3 | 1299-1319  1451-1473  1346-1374  1346-1362 |
| *RHOU*  Forward primer  Reverse primer  Reporter probe  Quencher probe | CAAGACGAGCCTGGTGG  GCTTGTCAAATTCATCCTGTC  CCTACTGCCTTCGACAACTTCTCCGC  TTGTCGAAGGCAGTAGG | NM_021205.5  NM_021205.5  NM_021205.5  NM_021205.5 | 849-865  983-1003  904-929  904-920 |
| *SPON2*  Forward primer  Reverse primer  Reporter probe  Quencher probe | GTGACCGAGATAACGTCCTC  GCGTTTCTGGAACTGAGG  CTGTCTACAATCTCATTGTCCCTGCTGGG  GGACAATGAGATTGTAGACAG | NM_012445.3  NM_012445.3  NM_012445.3  NM_012445.3 | 957-976  1135-1152  1104-1132  1112-1132 |
| *TDRD1*  Forward primer  Reverse primer  Reporter probe  Quencher probe | AAAGAAGTGAATATTAAGCCTG  TAGTAGGTCTGCTTGCACTG  ACGTCCTGCAAAATCAAAAAAACTAAACAA  TTTGATTTTGCAGGACGT | NM_198795.1  NM_198795.1  NM_198795.1  NM_198795.1 | 523-544  700-719  555-584  555-572 |
